# Shotgun metagenomic insights into secondary metabolite biosynthetic gene clusters reveal taxonomic and functional profiles of microbiomes in natural farmland soil

**DOI:** 10.1038/s41598-024-63254-x

**Published:** 2024-07-02

**Authors:** Bezayit Amare Kifle, Amsale Melkamu Sime, Mesfin Tafesse Gemeda, Adugna Abdi Woldesemayat

**Affiliations:** Department of Biotechnology, College of Biological and Chemical Engineering, Addis Ababa Science and Technology University, Addis Ababa, Ethiopia

**Keywords:** Antibiotics, Biosynthetic gene clusters, Secondary metabolites, Shotgun metagenomics, Soil microbiomes, Biotechnology, Computational biology and bioinformatics

## Abstract

Antibiotic resistance is a worldwide problem that imposes a devastating effect on developing countries and requires immediate interventions. Initially, most of the antibiotic drugs were identified by culturing soil microbes. However, this method is prone to discovering the same antibiotics repeatedly. The present study employed a shotgun metagenomics approach to investigate the taxonomic diversity, functional potential, and biosynthetic capacity of microbiomes from two natural agricultural farmlands located in Bekeka and Welmera Choke Kebelle in Ethiopia for the first time. Analysis of the small subunit rRNA revealed bacterial domain accounting for 83.33% and 87.24% in the two selected natural farmlands. Additionally, the analysis showed the dominance of Proteobacteria representing 27.27% and 28.79% followed by Actinobacteria making up 12.73% and 13.64% of the phyla composition. Furthermore, the analysis revealed the presence of unassigned bacteria in the studied samples. The metagenome functional analysis showed 176,961 and 104, 636 number of protein-coding sequences (pCDS) from the two samples found a match with 172,655 and 102, 275 numbers of InterPro entries, respectively. The Genome ontology annotation suggests the presence of 5517 and 3293 pCDS assigned to the “biosynthesis process”. Numerous Kyoto Encyclopedia of Genes and Genomes modules (KEGG modules) involved in the biosynthesis of terpenoids and polyketides were identified. Furthermore, both known and novel Biosynthetic gene clusters, responsible for the production of secondary metabolites, such as polyketide synthases, non-ribosomal peptide synthetase, ribosomally synthesized and post-translationally modified peptides (Ripp), and Terpene, were discovered. Generally, from the results it can be concluded that the microbiomes in the selected sampling sites have a hidden functional potential for the biosynthesis of secondary metabolites. Overall, this study can serve as a strong preliminary step in the long journey of bringing new antibiotics to the market.

Antibiotic resistance is one of the global challenges that demands immediate intervention. The problem of antibiotic resistance is not a new phenomenon. But recently, the number of microbes developing resistance to multiple drugs, different ecological locations affected by antibiotic resistance, and the emergence of hard-to-treat multidrug-resistant bacterial infections are accelerating at an alarming rate^1^. To combat the pressing issue of antibiotic resistance, the discovery of new antibiotics from microbes stands out as a notable measure. Microorganisms are renowned sources of secondary metabolites which in turn can be sources of antibiotics. Among the microbiomes in different ecological systems, the soil microbiome is known to be a prominent source of antibiotics.

The soil microbiome encompasses all the microorganisms, including archaea, bacteria, viruses, and fungi, that inhabit the soil environment. Despite the adversity they encounter in the soil environment, a diverse array of microorganisms thrives together, even within a single gram of soil^2^. The microbes in the soil environment are recognized to have a variety of functions in both natural and man-made ecological systems. Through studying soil microorganisms, microbiologists have come up with several important discoveries including the discovery of antibiotics and novel microbial metabolic pathways, such as nitrogen gas fixation and ammonia oxidation^3^. Empirical evidence suggests that the natural agricultural farmland soil environment harbors a greater abundance of taxonomic and phylogenetic richness, diversity, and heterogeneity of soil microbiota when compared to conventionally managed farming soil^4^. This is because natural agricultural farmland practices promote crop rotation, application of cover plants, and utilization of organic fertilizers instead of chemical ones. These practices are known for creating a favorable habitat for microorganisms to flourish. The intricate interplay between these microorganisms culminates in a concerted effort to compete for resources and to mount an effective defense against pathogens. Consequently, the microbiomes inhabiting the natural agricultural farmland soil environment can synthesize a range of secondary metabolites that are essential for maintaining soil health and enhancing productivity. Such products encompass a diverse array of compounds, including but not limited to antimicrobial agents and nutrient-cycling enzymes.

To have a complete understanding of the taxonomic profile and functional potential of microbiomes in the natural agricultural farmland soil environment and to gain an insight into their capacity for the production of novel secondary metabolites, relying solely on culture-dependent approaches is not adequate. This is because the majority of the microorganisms with an estimate of 99% are not accessible by traditional culture-dependent methods^2^. As a result, culture-independent techniques such as Shotgun Metagenomics approaches are needed not only to develop an understanding of the taxonomic and functional potential of microbiomes but also to gain insight into their capacity for the production of novel secondary metabolites^5^. Shotgun metagenomics involves, extracting the entire environmental DNA (eDNA) from the environmental sample, fragmenting the eDNA into smaller pieces, sequencing, and performing a lengthy downstream analysis for taxonomic and functional annotation of the microbiomes and for identifying novel BGCs for secondary metabolite biosynthesis. In the context of discovering novel BGCs responsible for the production of secondary metabolites, this approach is utilized, to characterize the biosynthetic potential of metagenomic samples, pinpoint promising targets, and facilitate the focused retrieval of complete biosynthetic pathways from eDNA cosmid libraries. Different studies have illustrated the application of shotgun metagenomics approaches for discovering novel BGCs for secondary metabolites from microbes^6,7^.

Soil microbes are recognized for their ability to produce secondary metabolites by expressing BGCs, which are composed of clusters of genes, encoding the specialized metabolic pathway enzymes for secondary metabolite biosynthesis. These secondary metabolites encompass a diverse array of chemical classes, including NRPS and PKS, which are known to have immense value in the medical sector owing to their ability to generate a wide array of antibiotics and immunosuppressive agents^8^. Previous studies have identified BGCs in abundance and performed further analysis on the “C” and “KS” domains from NRPS and PKS BGC classes respectively^8–10^. These studies have employed the Antibiotics and Secondary Metabolite Analysis Shell (AntiSMASH) pipeline to identify BGCs. Additionally, they utilize tools such as the second-generation Natural Product Domain Seeker (NaPDoS2) for further analysis, aiming to discover novel BGCs involved in secondary metabolites biosynthesis. Even though the studies performed taxonomic annotation and discovered the BGCs in abundance, they failed to give further information on the functional potential of the microbiomes in the selected sampling sites. Besides giving information about the taxonomic profile of the microbiomes and identification of BGCs by the application of tools such as AntiSMASH and NaPDoS2, the present study provides additional insight into the functional potential of the microbiomes by utilizing databases such as InterPro and KEGG. Two distinct natural agricultural farmlands have been selected as sample collection sites for this study. These farmlands were selected because they are maintained without the application of fire or synthetic chemicals, and are exposed to crop rotation practices. This makes them a suitable environment for achieving the objective of our research, which includes gaining an overall insight into the taxonomic composition and functional potential of the microbiomes and identifying novel putative Biosynthetic gene clusters for secondary metabolites biosynthesis.

## Results

### Physicochemical analysis

The results from physicochemical analysis consisted of organic carbon and total nitrogen, both measured in percentage (%); electrical conductivity (EC), measured in microsiemens per centimeter (µS/cm); total dissolved solids, measured in milligrams per liter (mg/L); temperature, measured in degrees Celsius (°C) and pH. The physical analysis results consisted of the percentage of sand, silt, and clay; the particle density, measured in grams per cubic centimeter (g/cm^3^); and the texture class of the soil samples. All the parameters were measured in triplicates and the mean and standard deviation were calculated as illustrated in Table 1 below.

**Table 1:** Physico-chemical analysis for soil samples with codes BNFC and BNFW.

Chemical analysis	BNFC Readings			Mean	SD	BNFW Readings			Mean	SD
	1	2	3			1	2	3		
O. Carbon (%)	3.361	3.353	3.371	3.361667	0.009018	2.501	2.402	2.602	2.501667	0.100002
T. Nitrogen (%)	0.331	0.322	0.342	0.331667	0.010017	0.271	0.262	0.281	0.271333	0.009504
EC (µS/cm)	130.01	129.02	131.01	130.0133	0.995004	260.02	259.01	261.01	260.0133	1.000017
pH	7.921	7.821	8.03	7.924	0.104532	8.161	8.152	8.171	8.161333	0.009504
TDS (mg/L)	68.01	67.02	69.01	68.01333	0.995004	120.01	119.02	121.01	120.0133	0.995004
T (°C)	18.01	17.03	19.01	18.01667	0.990017	24.01	23.02	25.02	24.01667	1.000017
Physical analysis										
% Sand	29.01	28.03	30.01	29.01667	0.990017	31.01	30.02	32.31	31.11333	1.148492
% Silt	39.01	38.031	40.01	39.017	0.989519	37.01	36.03	38.01	37.01667	0.990017
% Clay	32.01	31.02	33.01	32.01333	0.995004	32.01	31.011	33.03	32.017	1.009518
Textural class	Clay loam					Clay loam				
Particle density (g/cm^3^)	2.531	2.431	2.632	2.531333	0.1005	2.591	2.582	2.601	2.591333	0.009504

### Sequence data information summary

Illumina NovaSeq 6000 sequencing platform generated 7.2 Gb of data for sample BNFC and 7.8 Gb of data for sample BNFW. The metagenomic DNA sequence data information for both soil samples is summarized in Table 2.

**Table 2:** Metagenomic DNA sequence data information summary.

Sample	Raw reads	Raw data (Gb)	Effective (%)	Error (%)	Q20 (%)	Q30 (%)	GC (%)
BNFC	48162706	7.2	99.78	0.03	95.93	89.89	62.70
BNFW	46703900	7.8	99.80	0.03	95.41	88.59	63.46

### Quality control and FASTQC analysis results

After contaminant genome removal, 48,093,646 and 46,634,870 raw reads remained from sample BNFC and BNFW respectively.

### Metagenome assembly output

Quality Assessment Tool for Genome Assemblies, suggested that metaSPAdes assembler gave a superior output than the MEGAHIT assembler for both samples. metaSPAdes assembler generated 118865 contigs from sample BNFC. Contigs from sample BNFC had a maximum length of 8485 bp, an average length of 689.54 bp, and a minimum length of 500 bp. The GC content and AT content of the sample were 60.78% and 39.22%, respectively. For sample BNFW, metaSPAdes generated 70960 contigs. The contigs from this sample had a maximum length of 3689 bp, an average length of 667.57 bp, and a minimum length of 500 bp. The GC content and AT content of the sample were 62.58% and 37.42%, respectively.

### Taxonomic annotation

**Figure 1:**
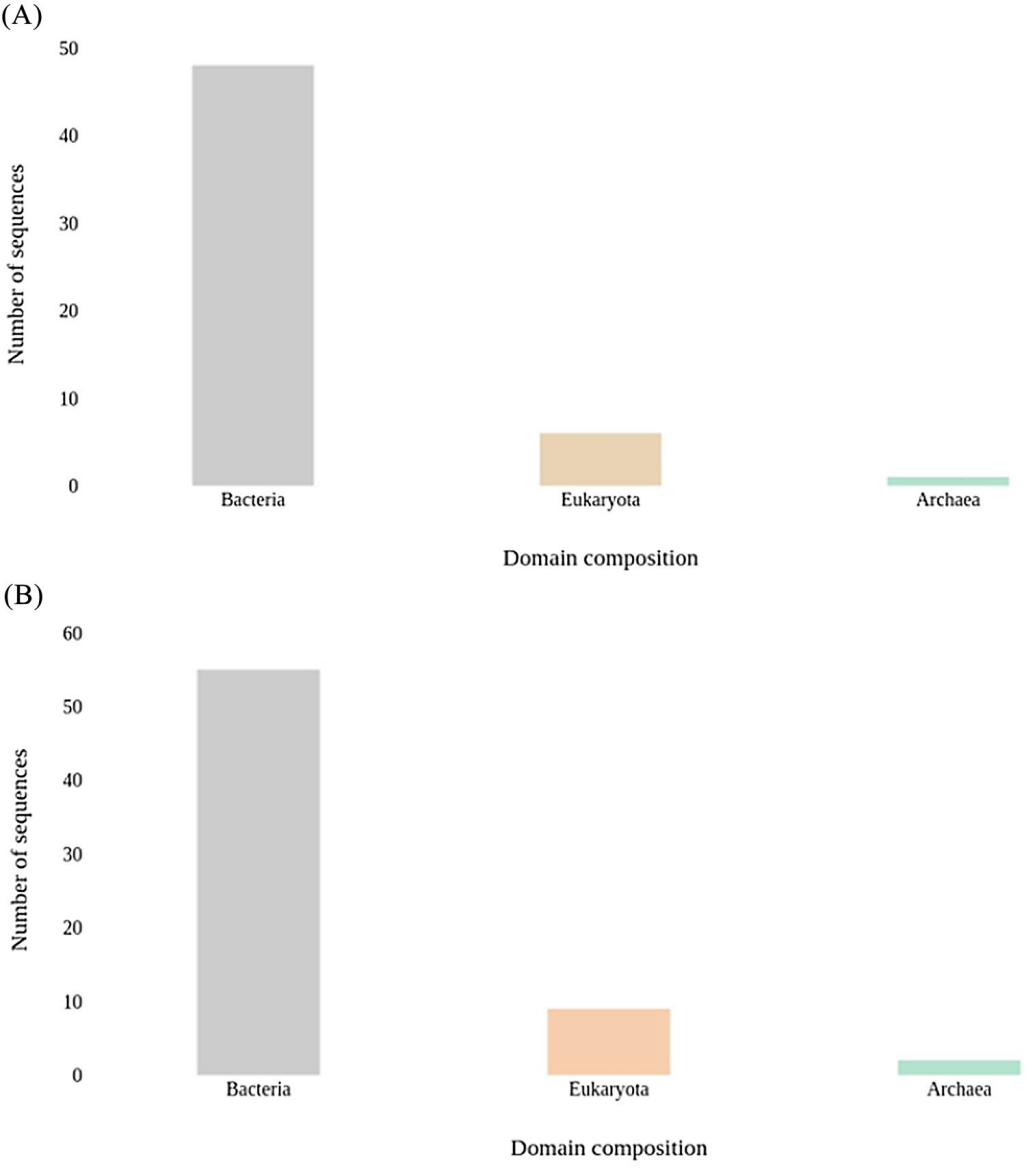
The domain composition of the samples as determined through SSU rRNA analysis. Sample BNFC (**A**) and sample BNFW (**B**). Bacteria is the dominant domain in both samples followed by Eukaryote and archaea domains.

Out of the assembled contigs, sample BNFC contained 864 contigs with predicted RNA and sample BNFW contained 644 contigs with predicted RNA. The taxonomic analysis of Small Subunit Ribosomal RNA (SSU rRNA) revealed the domain and phylum composition in both samples. The analysis unveiled Bacterial domain dominance in both samples, followed by Eukaryota and Archaea domains. Sample BNFC contained 48 contigs assigned to the Bacterial domain accounting for 87.24% moreover it encompassed 6 contigs assigned to the eukaryote domain representing 10.91%, and 1 contig assigned to the archaea domains making up 1.82%. Sample BNFW contained 55 contigs assigned to the Bacterial domain accounting for 83.33% in addition it included 9 contigs assigned to the eukaryote domain making up 13.64%, and 2 contigs assigned to the archaea domain representing 3.03% as indicated in Fig. 1.

**Figure 2:**
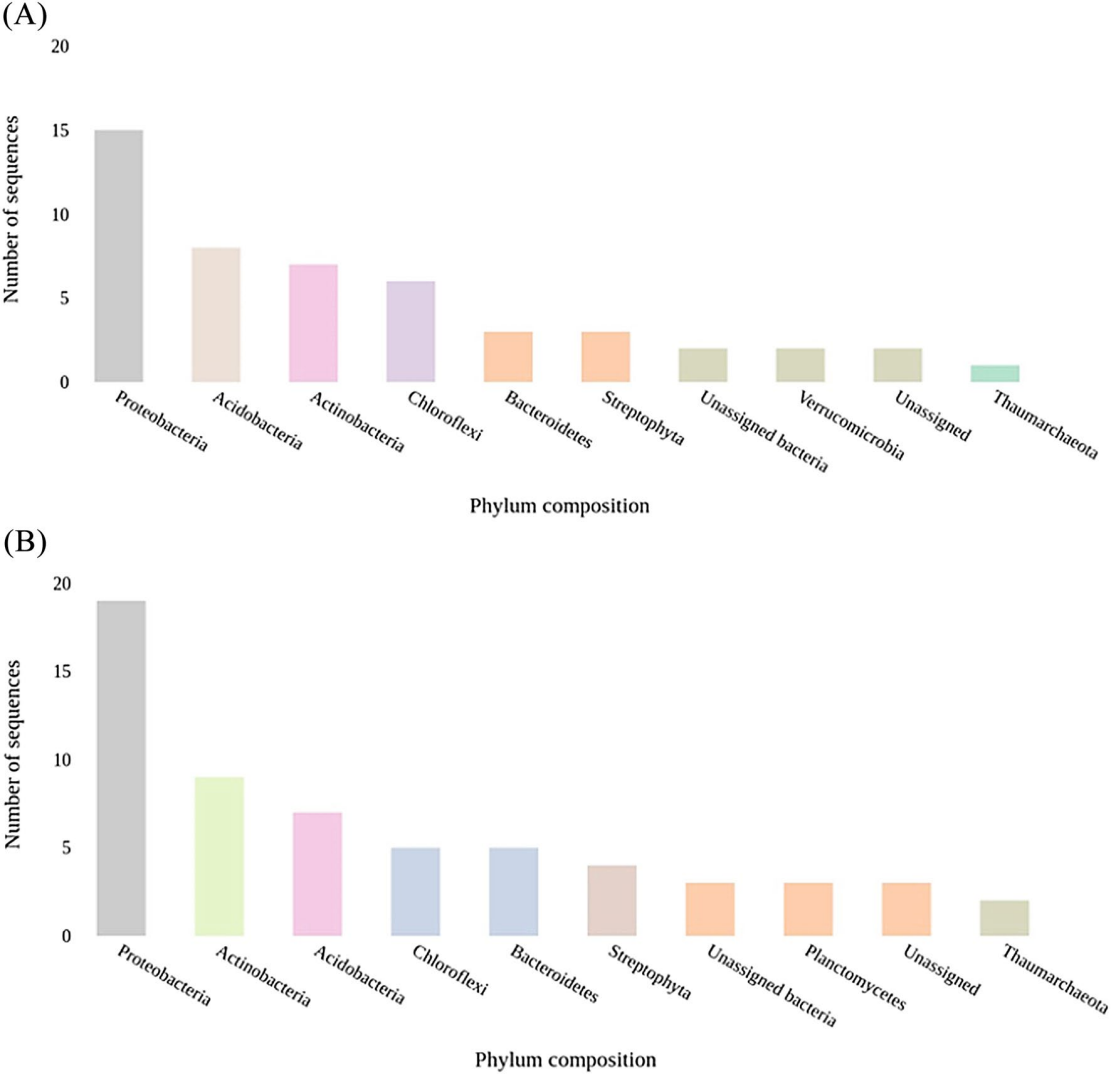
The Phylum composition of the samples determined through SSU rRNA analysis. Sample BNFC (**A**) and Sample BNFW (**B**). Proteobacteria, Actinobacteria and Acidobacteria were found to be the top three abundantly found phyla in both samples.

The analysis of the SSU rRNA also revealed the phylum composition of the microbiomes in both samples. For sample BNFC, as illustrated in Fig. 2A, the SSU rRNA analysis resulted in the phylum composition consisting of 15 contigs (27.27%) classified as Proteobacteria, 8 contigs (14.55%) as Acidobacteria, 7 contigs (12.73%) as Actinobacteria, 6 contigs (10.91%) as Chloroflexi, 3 contigs (5.45%) as Bacteroidetes, 3 contigs (5.45%) as Streptophyta, 2 contigs (3.64%) as Unassigned Bacteria, 2 contigs (3.64%) as Verrucomicrobia, 2 contigs (3.64% ) as Unassigned and 1 contig (1.82%) as Thaumarchaeota. For sample BNFW, as displayed in Fig. 2B, the SSU rRNA analysis resulted in the phylum composition consisting of 19 contigs (28.79%) classified as Proteobacteria, 9 contigs (13.64%) classified as Actinobacteria, 7 contigs (10.61%) classified as Acidobacteria, 5 contigs (7.58%) classified as Bacteroidetes, 5 contigs (7.58%) classified as Chloroflexi, 4 contigs (6.06%) classified as Streptophyta, 3 contigs (4.55%) classified as Unassigned Bacteria, 3 contigs (4.55%) classified as Planctomycetes, 3 contigs (4.55%) classified as Unassigned, 2 contigs (3.03%) classified as Thaumerchaeota.

### Gene prediction and functional annotation

**Figure 3:**
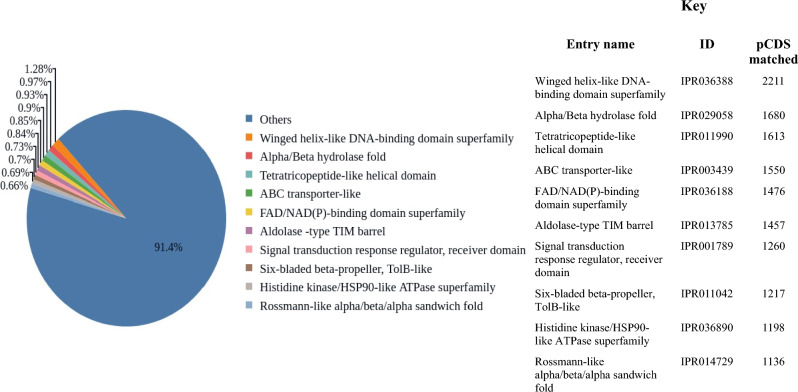
A summary of InterPro entry matches with descriptions for sample BNFC. The “Winged helix-like DNA-binding domain superfamily” InterPro entry experienced the highest number of matched CDSs, followed by the “Alpha/Beta hydrolase fold” InterPro entry”.

A total of 176961 Coding Sequences** (**CDS) from sample BNFC and a total of 104636 CDS from sample BNFW were predicted using FragGeneScan. The predicted CDSs were functionally annotated using InterProScan by querying the InterPro database which consists of Pfam, PRINTS, PROSITE, SUPERFAMILY, TIGRFAMs, Gene3d, PANTHER, PIRSR, HAMAP, SFLD, Coils, Gene ontology and AntiFam member databases. From the analysis, a total of 172655 InterPro matches based on the CDSs obtained from the sample BNFC and a total of 102275 InterPro matches from the CDSs originating from sample BNFW were found. See Fig. 3 below for sample BNFC and [see Supplementary Fig. 1] for sample BNFW which illustrates a summary of the top ten InterPro entry matches. “Winged helix-like DNA-binding domain superfamily” InterPro entry experienced the highest number of matched CDSs in both samples. From sample BNFC, 2211 protein-coding DNA sequences (pCDSs) matched this entry, and from sample BNFW, 1453 pCDSs matched this entry. “Alpha/Beta hydrolase fold” InterPro entry experienced the second-highest number of matched CDSs in both samples. 1680 pCDSs from sample BNFC and 1053 pCDSs from sample BNFW found a match to this entry.

**Figure 4:**
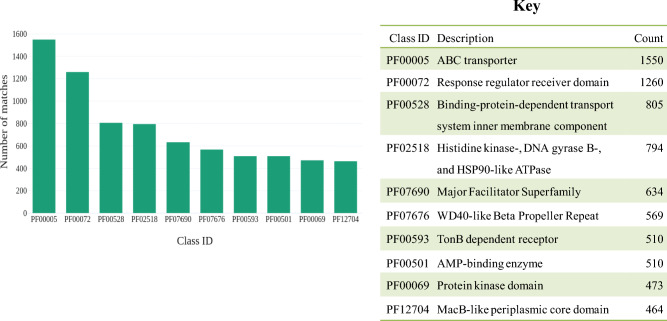
Top ten pfam entry match with their respective ID, description, and total pCDS count for sample BNFC. The “ABC transporter”, “Response regulator receiver domain”, and “Binding-protein-dependent transport system inner membrane component” are the top three pfam entries to which the highest number of pCDSs are assigned respectively.

When comparing the Pfam match results of the two samples, a greater proportion of similarity was observed. This is displayed in Fig. 4 for sample BNFC and [refer to Supplementary Fig. 2] for sample BNFW. The “ABC transporter” Pfam entry experienced the highest number of CDSs matches from both samples, with 1550 CDSs from sample BNFC and 914 CDSs from sample BNFW assigned to this Pfam entry.

It was also demonstrated that the “Response regulator receiver domain” is a Pfam entry to which 1260 pCDS from sample BNFC and 648 pCDS from sample BNFW were assigned. Once more, the “Binding-protein-dependent transport system inner membrane component”, Pfam entry had 805 pCDS from sample BNFC and 430 pCDS from sample BNFW assigned to it. “Histidine kinase-, DNA gyrase B-, and HSP90-like ATPase”, Pfam entry had 794 pCDS assigned from sample BNFC and 406 pCDS assigned from sample BNFW. Moreover, the “Major Facilitator Superfamily” Pfam entry had 399 pCDS from sample BNFC and 634 pCDS from sample BNFW assigned to it.

**Figure 5:**
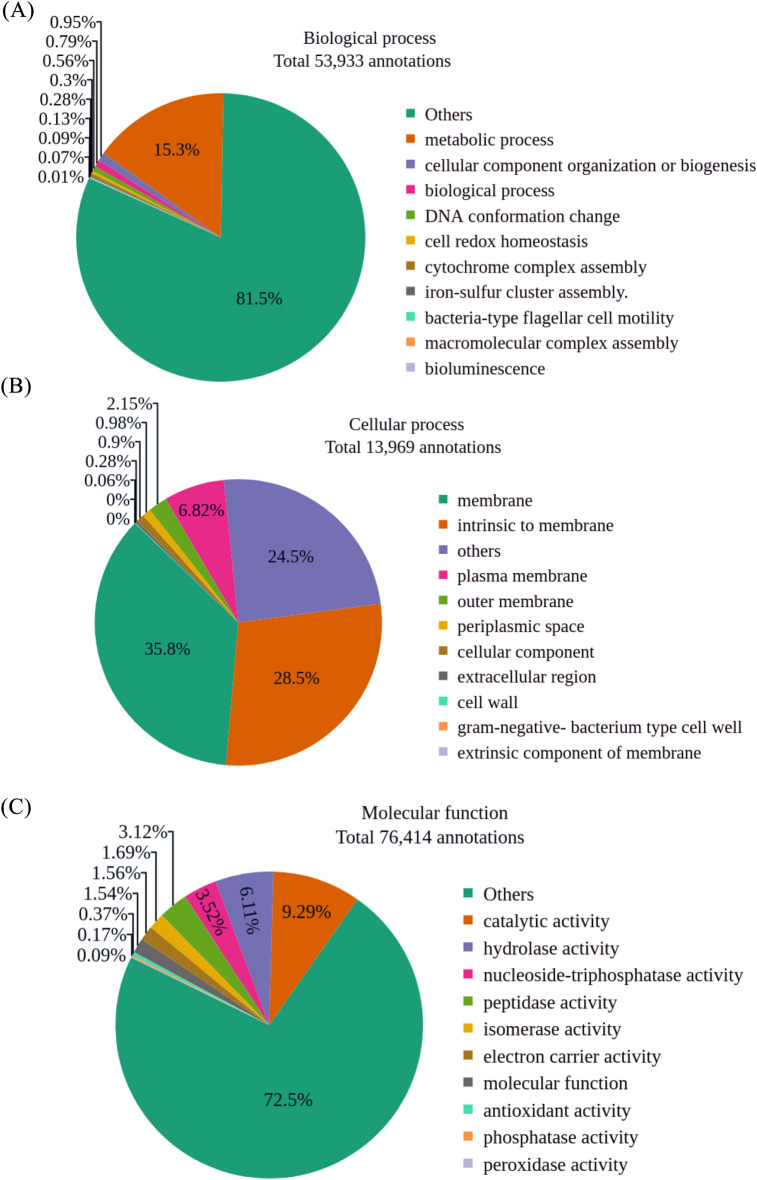
Sample BNFC GO terms annotations summary. There were 54933 Biological process annotations (**A**), 13969 Cellular component annotations (**B**), and 76414 Molecular function annotations (**C**).

The gene ontology (GO) term summary derived from the InterPro match for both samples includes annotations for Biological processes, Cellular components, and Molecular functions. Sample BNFC is associated with 54933 Biological process annotations, 13969 Cellular component annotations, and 76414 Molecular function annotations as illustrated in Fig. 5. Similarly, Sample BNFW comprises 33171 Biological Process annotations, 8367 Cellular Component annotations, and 45586 Molecular Function annotations, [see Supplementary Fig. 3].

### KEGG orthologs and pathway analysis

**Figure 6:**
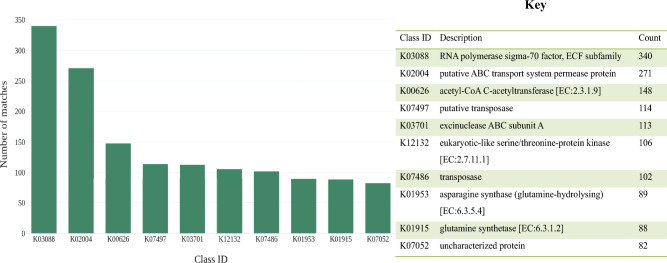
Top ten KO entries with their respective class ID, Description, and the number of pCDS assigned to them for sample BNFC. The “RNA polymerase sigma-70 factor, ECF subfamily”, “putative ABC transport system permease protein”, and “acetyl-CoA C-acetyltransferase [EC:2.3.1.9]” are the top three KO classes to which the highest number of pCDSs are assigned respectively.

The KAAS server was used to annotate the translated gene sequences by using the GHOSTX search tool and SBH method. Different ortholog groups with unique identifiers annotated the query sequences based on their functional and evolutionary similarity. A summary of the top ten identified KO groups with their respective KO numbers and descriptions are displayed in Fig. 6 for sample BNFC and [see Supplementary Fig. 4] for sample BNFW.

**Figure 7:**
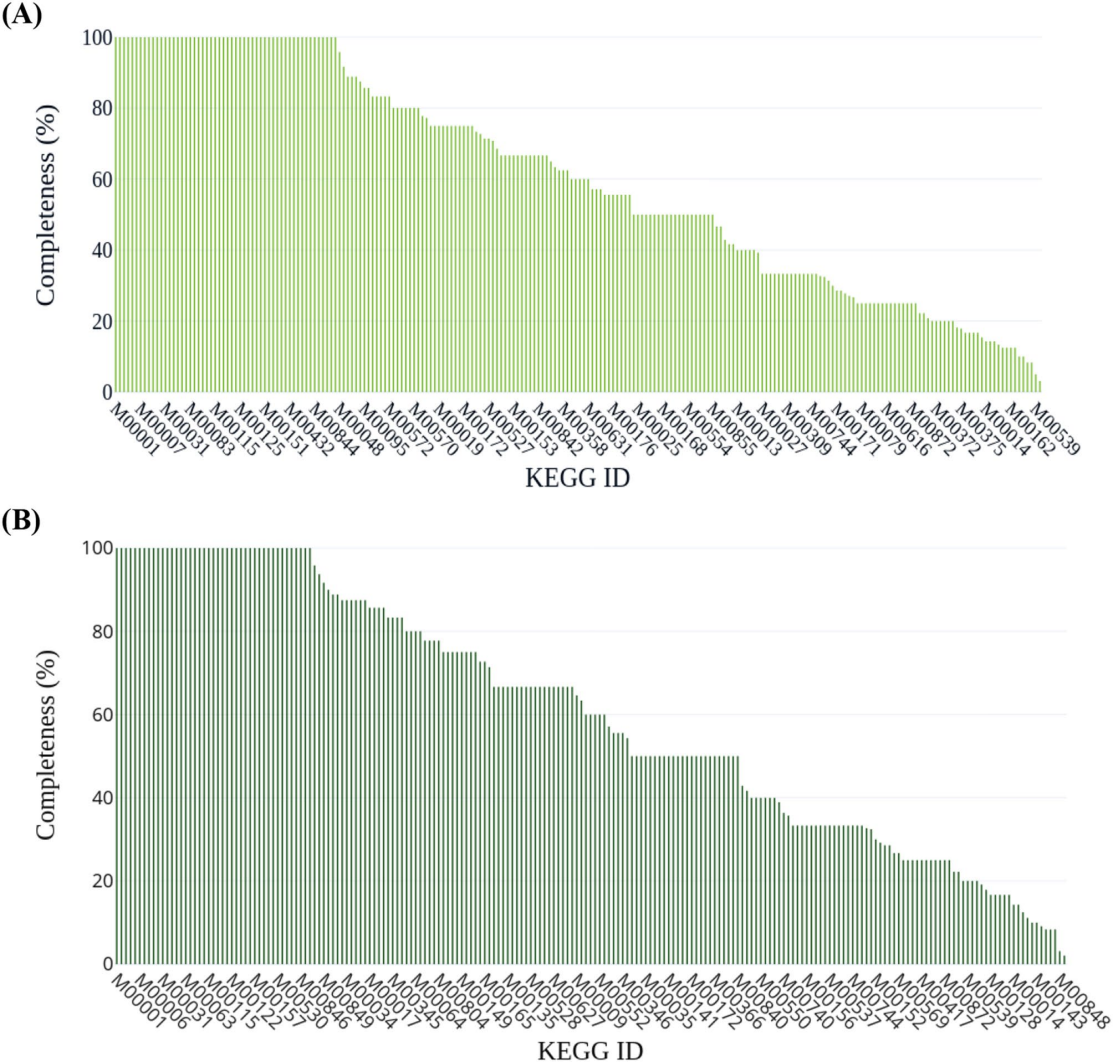
KEGG modules categories with their respective accessions and relative completeness for sample BNFC (**A**) and sample BNFW (**B**). According to the annotation the sequence data set from sample BNFC contained 225 identified KEGG modules with 55 of them fully complete and sample BNFW contained 208 identified KEGG modules with 44 of them fully complete.

According to the annotation performed by using the KAAS server, the sequence data set from sample BNFC contained 225 identified KEGG modules and the sequence data from sample BNFW contained 208 identified KEGG modules in total. Sample BNFC contained 55 fully complete KEGG modules among the identified modules and sample BNFW contained 44 fully complete KEGG modules among the total identified modules. Figure 7 illustrates the identified KEGG modules with their accessions and relative completeness for both samples. Full information on the fully completed KEGG modules can be found for sample BNFC [see Supplementary Table 1] and for sample BNFW [see Supplementary Table 2].

### Prediction of biosynthetic gene clusters (BGCs)

**Figure 8:**
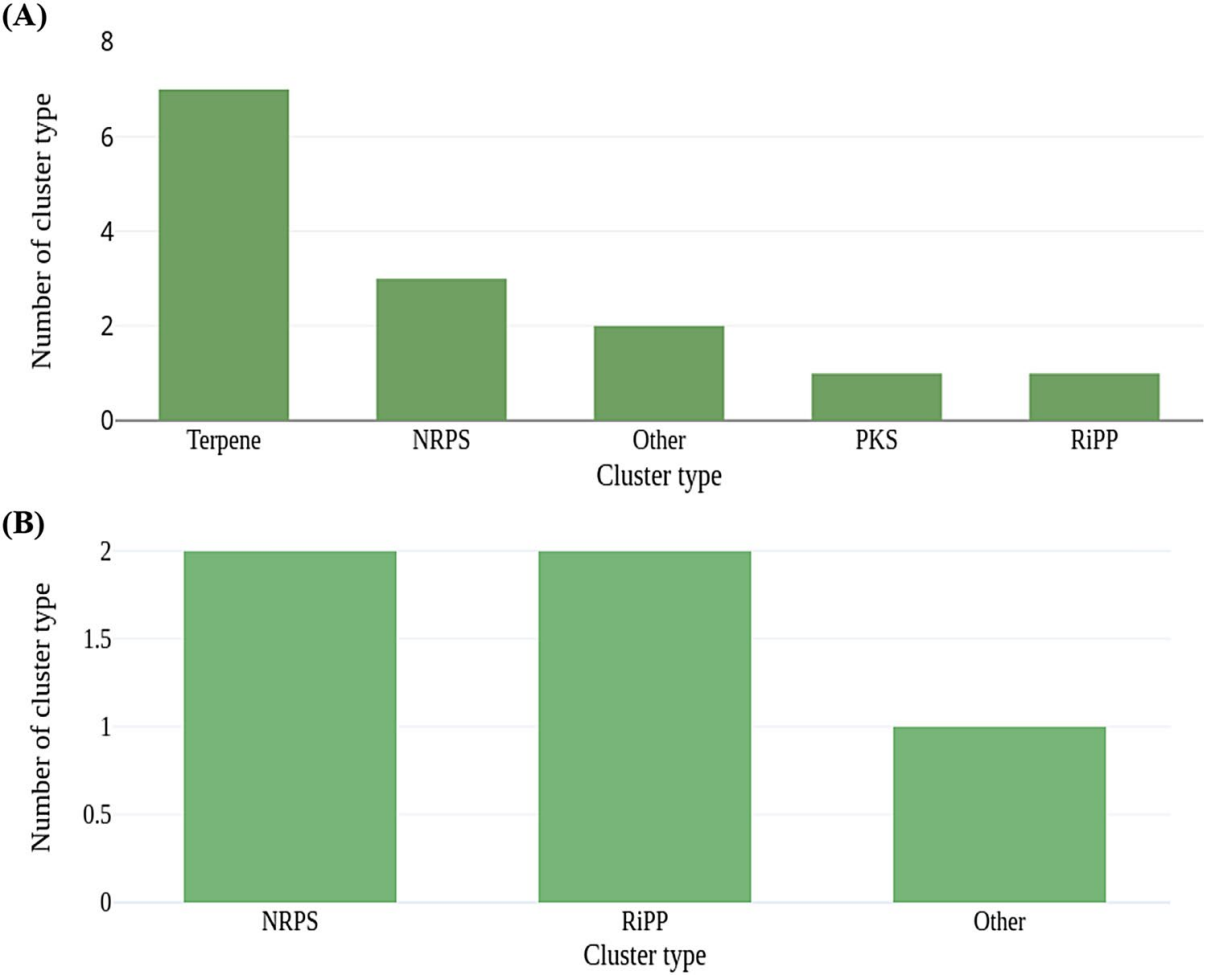
Predicted BGCs for sample BNFC (**A**) and BNFW (**B**) along with their cluster type and respective count. Terpenes, NRPS, Others, PKS, and RiPP BGCs are the predicted BGCs from both samples.

AntiSMASH tool predicted 7 Terpenes, 3 NRPS, 2 Others, 1 PKS, and 1 Ripp BGCs from sample BNFC and 2 NRPS, 2 Ripp, and 1 Other BGCs from sample BNFW as displayed in Fig. 8A and B, respectively.

**Figure 9:**
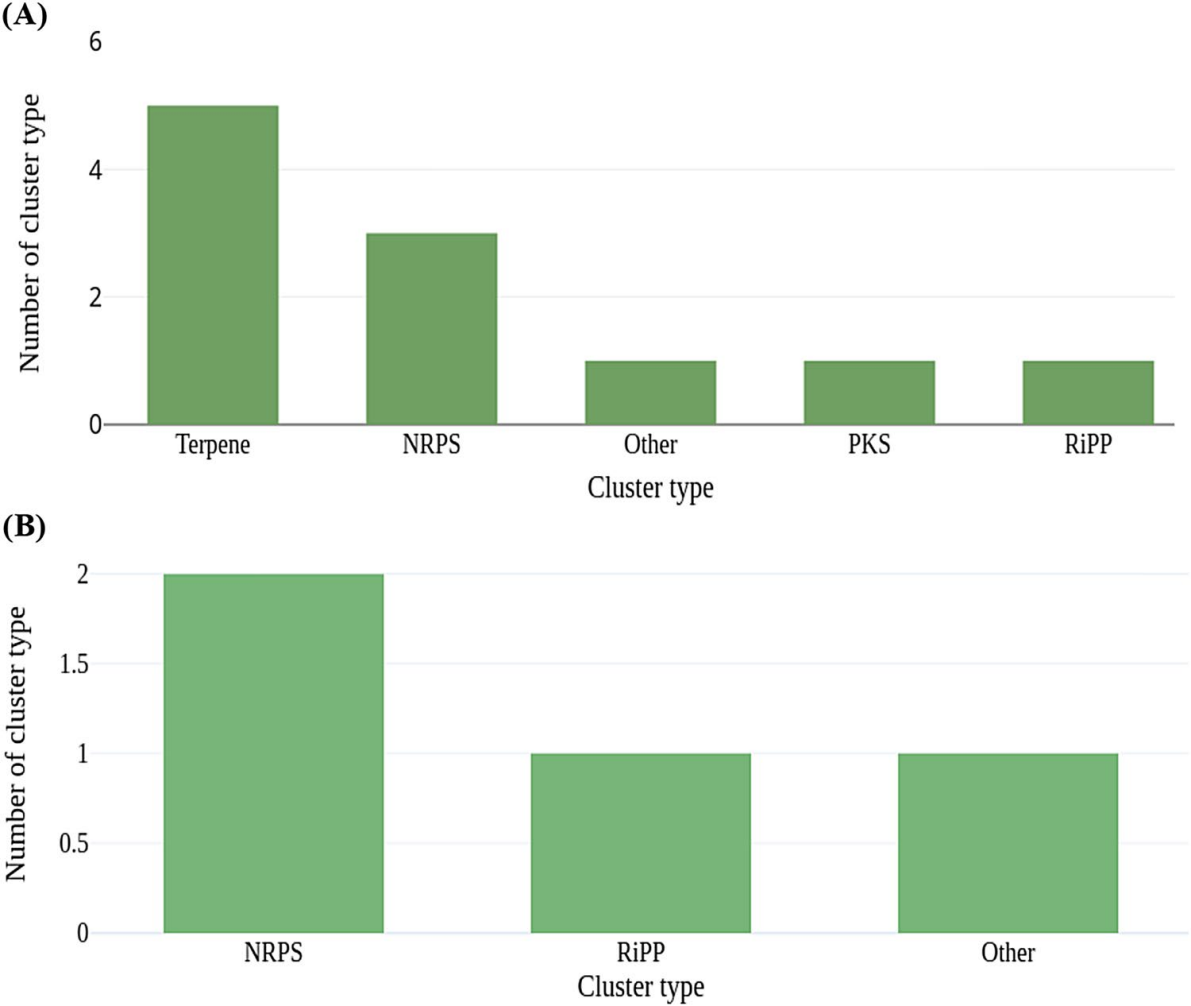
Predicted BGCs having no match with MIBiG repository for sample BNFC (**A**) and BNFW (**B**) along with their respective count and classification. Among the predicted BGCs all the NRPS, PKS, and Ripp BGCs two of the Terpenes and one of the Other BGCs did not find a match in the MIBiG repository.

Comparing the identified BGCs to BGCs with experimentally determined secondary metabolites in the MIBiG repository revealed that clusters such as Terpenes and others found a match in the repository as indicated in Table 3 whereas clusters such as NRPS, PKS, and Ripp found no match in the repository as illustrated in Fig. 9A for sample BNFC. For the sample BNFW, only one Ripp cluster found a match in the MIBiG repository as indicated in Table 4 on the other hand, clusters such as NRPS and others received no match in the MIBiG repository as illustrated in Fig. 9B.

**Table 3:** BNFC antiSMASH Predicted BGCs with MIBiG repository entry match. Two of the terpene and One of the “Other” BGCs found a match with BGCs in the MIBiG repository responsible for the production of the compounds Hopene, Carotenoid, and Amylocyclicin, respectively.

BGC accession	Similarity score	Type	Compound	Phylum	Species
BGC0000663	0.06	Terpene	Hopene	Actinobacteria	*Streptomyces coelicolor*
BGC0000633	0.12	Terpene	carotenoid	Actinobacteria	*Streptomyces avermitilis*
BGC0000616	0.16	Other	Amylocyclicin	Firmicutes	*Bacillus velezensis*

**Table 4:** BNFW AntiSMASH Predicted BGCs with MIBiG repository entry match. The Ripp BGC from the sample found a match with BGC in the MIBiG repository responsible for the production of the compound Mildiomycin.

BGC accession	Similarity score	Type	Compound	Phylum	Species
BGC0000882	0.15	Ripp	Mildiomycin	Actinobacteria	*Streptomyces rimofaciens*

### Identification of putative novel BGCs for secondary metabolites

**Figure 10:**
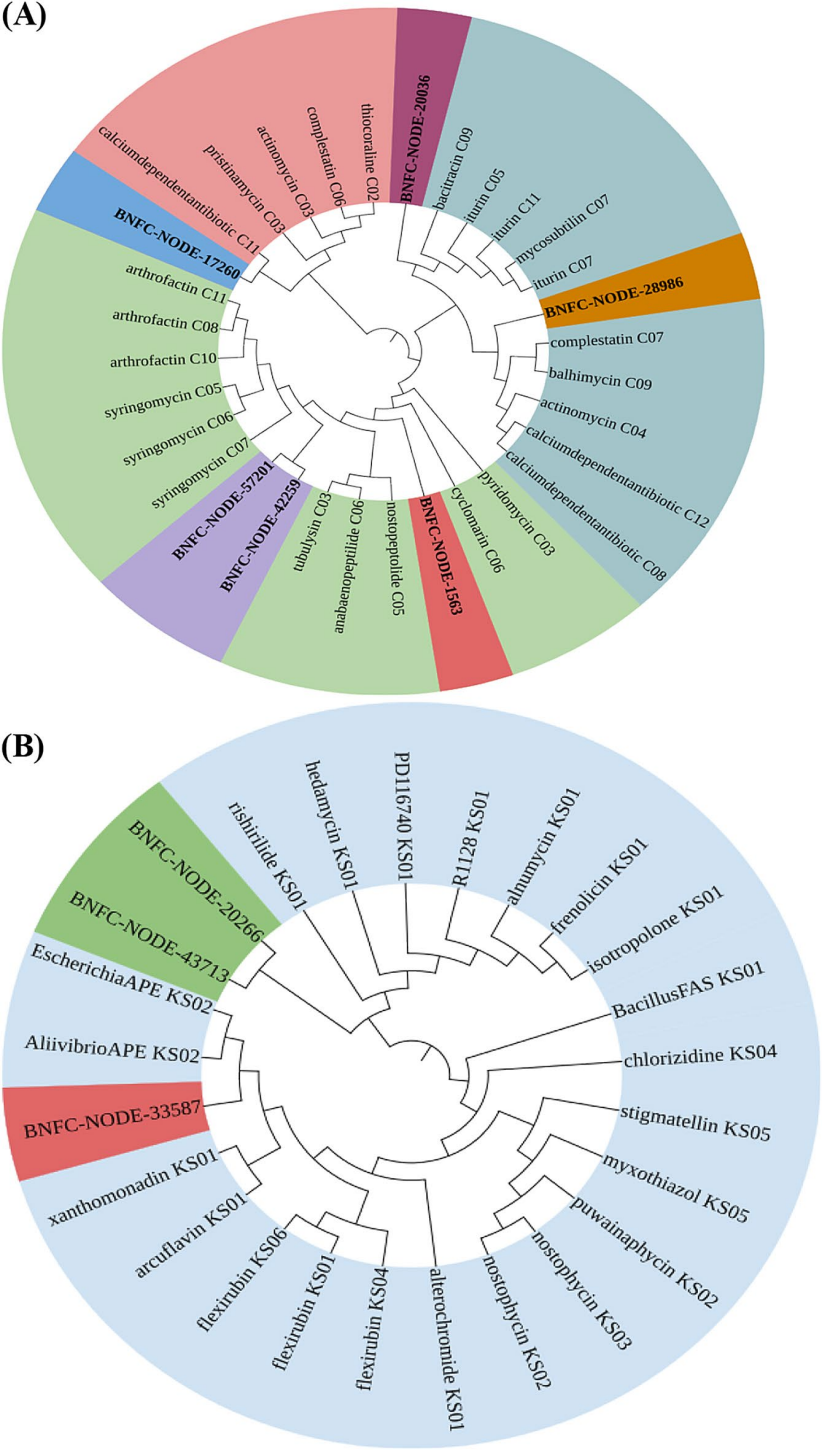
NaPDos C domain analysis result (**A**) and KS domain analysis result (**B**) for sample BNFC. The six “C” domains originated from sample BNFC (**A**) had a variably unique product as they have a percent identity of less than 85 with products such as pyridomycin, pristinamycin, artirofactin, calcium dependent antibioic, syringomycin, and mycosubtilin. Similarly, the three “KS” domains from sample BNFC (**B**) also had a percent identity of less than 85 with products such as hedamycin, Aliivibrio fischen aryl polyene, and rishirlide.

**Figure 11:**
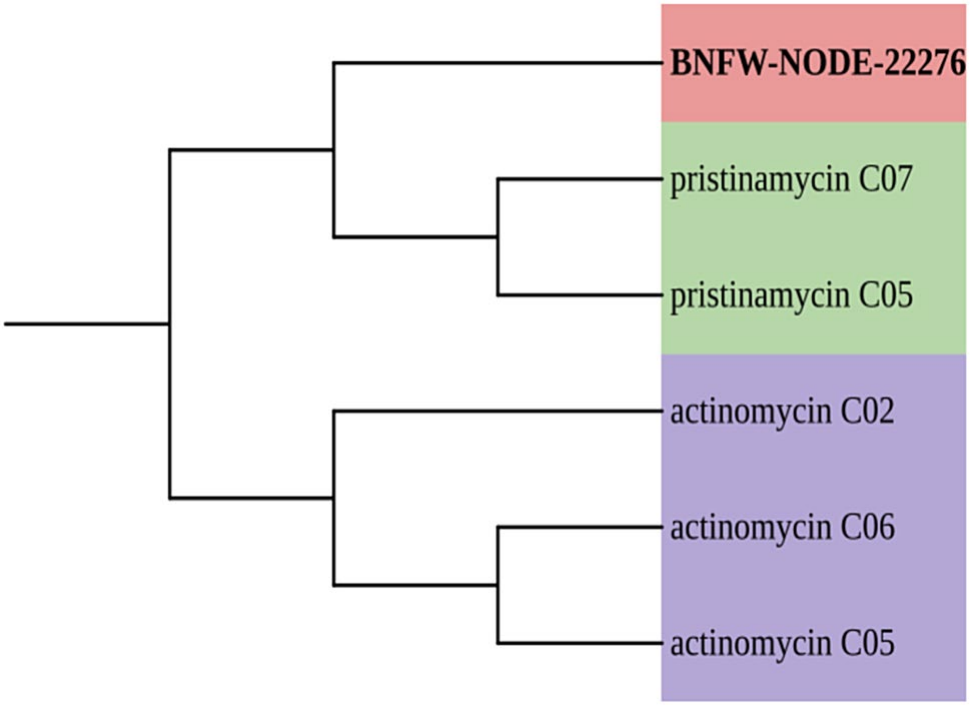
NaPDos C domain analysis results for sample BNFW. the identified “C” domain in sample BNFW had a percent identity of 62 with the product pristinamycin.

The analysis result of NaPDoS2 visualized by iTol^11^ is displayed in Fig. 10 for the “C” and “KS” domains of sample BNFC and in Fig. 11 for the “C” domain of sample BNFW. When comparing all the six “C” domains originated from sample BNFC, it was evident that they had a variably unique product as they have a percent identity of less than 85 such that BNFC-NODE-1563, BNFC-NODE-17260, BNFC-NODE-42259, BNFC-NODE-28986, BNFC-NODE-57201, and BNFC-NODE-20036 had a percent identity of 37, 44, 45, 45, 37 and 32 with the product pyridomycin, pristinamycin, artirofactin, calcium-dependent antibiotic, syringomycin and mycosubtilin respectively. The 3 identified “KS” domains from sample BNFC also had a percent identity of less than 85 with varying products such that BNFC-NODE-20266, BNFC-NODE- 33587, and BNFC-NODE-43713 had a percent identity of 43, 46, and 43 with the product identified as hedamycin, Aliivibrio fischen aryl polyene and rishirlide. Similarly, the identified “C” domain in sample BNFW located on BNFW-NODE-22278 had a percent identity of 62 with the product pristinamycin.

Further analysis of the Terpene BGCs by querying them on the NCBI Metagenomics proteins (env nr) database utilizing the BLASTn algorithm resulted in two of the terpene BGCs located on BNFC-NODE-5111 and BNFC-NODE-7202 found a match with squalene–hopene cyclase and Lycopene cyclase proteins respectively. The remaining terpene BGCs which were located on BNFC-NODE-2295, BNFC-NODE-2318, BNFC-NODE-4329, BNFC-NODE-9530, and BNFC-NODE-9609 resulted in hypothetical protein match with different percent identity as indicated on Table 5 above.

**Table 5:** The first top hit for each Terpene BGC in the NCBI Metagenomics proteins (env nr) database and the first top hit for each Ripp BGC in the NCBI Metagenomics proteins (env nr) database.

Cluster type	Description	E-value	Percent identity (%)
Terpene	squalene–hopene cyclase	4e − 137	54
	Lycopene cyclase protein	1e − 73	42
	hypothetical protein	1e − 65	38
	hypothetical protein	3e − 15	60
	hypothetical protein	5e − 89	43
	unannotated protein	6e − 05	42
	uncharacterized protein	2e − 27	68
Ripp	coenzyme PQQ synthesis protein D	2e − 16	40
	hypothetical protein	1e − 99	53

The analysis of Ripp BGCs by querying them on the NCBI Metagenomics proteins (env nr) database for sample BNFW resulted in one of the Ripp located on BNFW-NODE-2144 matched with coenzyme PQQ synthesis protein D with a percent identity of 40. The Ripp located on BNFW-NODE-1601 found no match with the known protein as illustrated in Table 5 above.

## Discussion

### Soil physicochemical property and taxonomic annotation

The physicochemical analysis results indicated a high organic carbon content, a slightly alkaline pH, and a moderate temperature range for both samples. A recent study has suggested that the high organic content of the soil samples provides a favorite environment for microbial growth, metabolism, and nutrient cycling^12^. The soil texture results classified both samples as clay loam. This texture class indicates a mixture of different particle sizes which gives a balanced soil texture, as a result, the texture class provides a suitable environment for microbial colonization, nutrient solubility, and nutrient availability^13^. Overall from the physicochemical analysis result, it can be concluded that both the sampling sites support the growth of diverse microbial domains and phyla, which in turn, might have functional potential for the production of unique secondary metabolites^14^.

SSU rRNA analysis revealed the dominance of the bacterial domain in both samples. Sample BNFC had 87.24% of the contigs assigned to the bacterial domain, and sample BNFW had 83.33% of the contigs assigned to the bacterial domain. This illustrates that the majority of the contigs generated in both samples were assigned to the bacterial domain. This result is supported by previous studies that have indicated bacteria to be dominant in various agricultural soil environments^15–17^.

The phylum composition in both samples is also illustrated by SSU rRNA analysis, an additional table file shows this in more detail for sample BNFC [see Supplementary Table 3] and for sample BNFW [see Supplementary Table 4]. Proteobacteria was found to be the dominant phylum in both samples. The abundance of proteobacteria in the soil samples aligns with other previous studies that pointed out the high prevalence of proteobacteria in various agricultural soil environments^18,19^. Proteobacteria is an extremely diversified phylum responsible for the production of a wide range of bioactive products; including antibiotics, antifungal and antitumor agents^20^. The dominance of proteobacteria in this study can be related to the potential of this phylum for secondary metabolite production and its fundamental ecological role. Actinobacteria a well-recognized phylum, for secondary metabolite biosynthesis was also abundantly identified in both samples. A study on agricultural soil of maize plants has reported an abundance of the actinobacteria phylum together with other phyla which supports the present study^16^. The availability of this phylum in large proportion in the microbial communities of both samples implies the potential of the microbes for the production of a wide range of secondary metabolites in these environments. The other abundantly identified phylum in both samples was Acidobacteria. According to the analysis result this phylum was more abundant in sample BNFC than in sample BNFW. This variation in abundance might be credited to the fact that sample BNFC is collected from the agricultural soil of maize plants, including both rhizospheric and bulk soil samples. This is supported by a study that demonstrated the dominance of Acidobacteria in samples collected from the rhizospheric soil of maize plants^17^. Even though the phylum Acidobacteria is relatively less explored for the production of natural products by previous studies, this study can be used as an initial point for gaining an insight into their functional capability. Moreover, from the analysis, the identification of unassigned phyla in a significant proportion is a fascinating finding. These unassigned phyla represent poorly understood and classified groups of microbes holding immense potential for novel microbial diversity and functional capabilities, including the production of novel natural products.

### Functional annotation

The results from the database InterPro^21^ suggested that, the majority of the predicted coding sequences (pCDSs) from the studied samples matched with the “Winged helix-like DNA-binding domain superfamily” InterPro entry. This entry consisted of DNA-binding proteins sharing a common structural motif^22^. Proteins with this domain can be classified as transcriptional factors which can be associated with the biosynthesis of secondary metabolites such as antibiotics, as regulatory elements play a substantial role in the expression of genes within BGC^23^. Plenty of pCDSs from both studied samples found a match with the “Alpha/Beta hydrolase fold” InterPro entry which consisted of hydrolytic enzymes with α/β-sheet of eight β-sheets connected by α-helices enzyme core in common among all the enzymes^24^. The presence of enzymes with hydrolytic properties can be associated with the modification, arrangement, and processing of molecules that are utilized as precursors for natural product assemblage. “FAD/NAD(P)-binding domain superfamily” experienced high pCDSs matches from sample BNFC and a reasonably comparable number of pCDSs matches from sample BNFW. During redox reaction, this domain functions as an electron binding and transferring region, and the occurrence of this domain in both studied samples might suggest the availability of enzymes, that utilizes FAD or NAD(P) as cofactors. These enzymes are commonly needed in different biosynthetic processes including in those pathways that are involved in the biosynthesis of secondary metabolites. On the other hand, a high number of pCDSs from both studied samples that matched the “ABC transporter-like” Interpro entry have functional implications that are related to the transportation of different molecules across cellular membranes. The availability of “ABC transporter-like domain” in both samples may suggest the presence of transport systems essential for the biosynthesis of natural products and their exportation^25^. Similarly, a great number of pCDSs in both samples matched the “Aldolase-type TIM barrel" InterPro entry that consisted of class I aldolases which contain the TIM barrel domain as exposed by X-ray crystallography study^26^. Class I aldolases, catalyze the formation of the Carbon–Carbon bonds and are involved in the formation of complex carbon frameworks localized in natural products. The availability of these enzymes in both samples may imply, the presence of biosynthetic enzymes that have a function in the assembly of components for the biosynthesis of natural products.

The results from the extracted Pfam database suggest that the “ABC transporter” had the highest number of pCDSs matches from both samples. This entry consisted of about 3 M proteins that are involved in the transportation of different types of compounds across biological membranes. “ABC transporters” frequently take part in the translocation of secondary metabolites precursors, intermediates, and final products across cell membranes. Interestingly, a recent study made a report on the ABC transporter that constitutes an essential part of the nonribosomal peptide biosynthetic machinery for the biosynthesis of secondary metabolites^27^. Another study related the ABC transporter with the maturation of the natural product Lasso Peptide Cochonodin I synthesized from the Ripp BGC^28^. Consequently, the abundant presence of these proteins in both samples signifies the considerable functional potential of the microbial community within the samples concerning secondary metabolites biosynthesis. Similarly, several pCDSs from both samples found a match with the “Major Facilitator Superfamily” Pfam entry which constituted of 2 M membrane proteins that represent the largest family of secondary transporters in all life forms. These proteins include transporters that can function by translocating natural products precursors, intermediates, and final products across the cell membrane. A recently published study focused on characterizing the “Major Facilitator Superfamily Transporters” associated with the antibacterial Pantoea natural product^29^. Therefore the availability of these proteins in both samples can be a strong indication for the presence of microbes with secondary metabolites production capacity. Furthermore, many pCDSs from both samples found a match to the Pfam entry, “TonB-dependent receptor” (TBDR) that comprised 473 K proteins. TBDRs mediate substrate-specific transport across the outer membrane by employing energy derived from the proton motive force, transmitted from the TonB complex located in the inner membrane^30^. During the biosynthesis of secondary metabolites, bacterial cells may need to uptake molecules such as; minerals, vitamins, aromatic compounds, and plant-derived compounds that are then imported by the TonB receptors^30^. Another study reported that TonB Dependent Receptor regulates the biosynthesis of antifungal secondary metabolites in bacterial cells^31^. To the Pfam entry, “MacB-like periplasmic core domain” several pCDSs from both samples were matched. This entry consisted of 206 K proteins. The periplasmic core domains localized in a variety of ABC transporters are represented by this protein family entry. The MacB-like proteins have functions related to the efflux system of the cell, which involves the removal of toxic molecules from the cell. In addition, the MacB-like periplasmic proteins may be involved in the exportation of secondary metabolites such as antibiotics from the cell^32^. The presence of these proteins in both samples is a piece of strong evidence that illustrates the production of secondary metabolites such as antibiotics by the soil microbiomes in the selected sampling sites.

The GO slim annotation results of both samples represent generalized, high-level functional categories across the three GO term classifications. In terms of biological process annotation, a significant number of pCDSs from both samples were assigned to the GO term “metabolic process.” This broad GO slim term encompasses the chemical reactions and pathways involved in living organisms. within the context of secondary metabolites biosynthesis, this GO term includes the key biological processes such as the synthesis of precursor molecules and the enzymatical conversion of these molecules to produce complex secondary metabolite compounds. Notably, a more specific complete GO term annotation of sample BNFC [see Supplementary Table 5] and for sample BNFW [see Supplementary Table 6] revealed an intriguing finding. Specifically, 5517 pCDSs from sample BNFC and 3293 pCDSs from sample BNFW were assigned to the “biosynthetic process” GO term, which is directly linked to secondary metabolite biosynthesis. Another GO slim term for a biological process that may be relevant to secondary metabolite biosynthesis is “DNA conformation change.” This term refers to the cellular process involving the structural conformational change of DNA molecules. Configurational change in DNA molecules can be associated with the transcriptional regulation of genes within BGCs, thereby determining the biosynthesis of secondary metabolites. The “cell redox homeostasis” GO slim term for biological process encompasses all the biological processes that maintain the redox environment within the cell and its organelles. The maintenance of a balanced environment within the cell is crucial for the proper functioning of enzymes and pathway reactions involved in the biosynthesis of secondary metabolites. The “cytochrome complex assembly” and “iron-sulfur cluster assembly” GO terms for biological processes describe enzymatic reactions that involve electron-carrying or redox-active molecules. Redox-active molecules and cofactors play a crucial role in secondary metabolite biosynthesis, serving as an electron transfer mechanism and potentially requiring specific cofactors in the biosynthesis process. The classification of cellular component GO terms mainly consisted of terms associated with membrane components such as, “membrane”, “Intrinsic to membrane”, “plasma membrane”, and “outer membrane.” As supported by previous studies, membrane-associated regions are a potential location for secondary metabolite biosynthesis in microbial systems^33,34^. These regions function by regulating, binding, and transporting enzymes involved in the biosynthesis of secondary metabolites. The cellular component GO term “extracellular region” is used to annotate gene products that are located outside of the outermost layer of the cell. Unlike membrane-associated components, these gene products are not attached to the surface of the cell. A previous study has associated extracellular gene products that function as signaling molecules for regulatory proteins, with the expression of secondary metabolite biosynthesis gene-cluster^35^. Consequently, the availability of these gene products in both samples can be associated with the regulation of BGCs for secondary metabolites biosynthesis. For molecular function GO slim annotation, most of the pCDSs of both samples are annotated with the “catalytic activity” GO term. This general GO term describes the activity of naturally occurring enzymes. Enzymatic activity can also be described by GO terms such as; “hydrolase activity” which involves the hydrolysis of bonds including peptides, esters, and glycosidic, and “isomerase activity” which involves the rearrangement of bonds in molecules. In secondary metabolite biosynthesis, enzymes with various and intricate catalytic activities play an essential role^36^. The availability of pCDSs with different enzymatic activity in both samples can be considered the major indicator of the potential of the microbes in both samples for secondary metabolite biosynthesis.

### KEGG orthologs and pathway analysis

The analysis resulted from the KAAS server revealed the dominance of the ortholog group “RNA polymerase sigma-70 factor, ECF subfamily” in both samples to which a reasonable number of pCDSs were annotated. This gene-product group consisted of sigma-70 factor proteins which are primary sigma factors associated with bacterial RNA polymerase. Specifically, this entry consisted of the Extra Cytoplasmic Function (ECF) subfamily proteins which function by responding to environmental cues such as stress. These groups of proteins play an important role by initiating gene expression through the process of transcription by guiding RNA polymerase to the promoter region of the DNA sequence. The presence of this protein group in both samples can strongly suggest the potential of the microbiomes for secondary metabolite biosynthesis, as they are involved in the transcription activation process of BGCs. A previous study has related this group of proteins with metabolic pathways in *Streptomyces* spp^37^, which are known for secondary metabolite biosynthesis specifically antibiotics. There is a functional association of a considerable number of pCDSs originating from the investigated samples to an ortholog group “acetyl-CoA C-acetyltransferase which as an entry represents one of the key enzymes in the mevalonate pathway^38^. The mevalonate pathway is known for the biosynthesis of isoprenoids and polyketides, which are well-known classes of secondary metabolites. The presence of this KO entry in both samples might suggest the availability of microbes with the potential for the production of bioactive molecules such as; isoprenoids and polyketides.

The result from the KEGG module database revealed the abundance of KEGG modules for the biosynthesis of terpenoids and polyketides in both samples. Among the identified KEGG modules, the ones which have 100% or near 100% completeness that represent a pathway module involved in the Biosynthesis of terpenoids and polyketides and Terpenoid backbone biosynthesis were observed to be “C5 isoprenoid biosynthesis, non-mevalonate pathway “ (ID: M00096), “C10-C20 isoprenoid biosynthesis, bacteria” (ID: M00364) and “C5 isoprenoid biosynthesis, mevalonate pathway” (ID: M00095) while a pathway module involved in the Biosynthesis of terpenoids and polyketides and Polyketide sugar unit biosynthesis was represented by “dTDP-L-rhamnose biosynthesis” (ID: M00793). Terpenoids and polyketides are well-recognized secondary metabolites. Terpenoids are a diverse class of organic compounds primarily synthesized by plants and consisting of Isoprene as their building block. Even though, bacteria are not well-recognized sources of Terpenoids, a recent advancement in genomics is revealing the genomic potential of bacteria for terpenoid biosynthesis^39^. In contrast, polyketides are a prominent source of bacterial secondary metabolite. These complex and diverse organic compounds primarily exhibit antimicrobial properties and are renowned sources of antibiotics^40,41^. In both samples, the abundant occurrence of KEEG modules for terpenoids and polyketide biosynthesis strongly suggests the presence of microorganisms with potential capabilities for the biosynthesis of these secondary metabolites. KEEG Pathway modules for the biosynthesis of other secondary metabolites were also identified. Some of these modules include “Rebeccamycin biosynthesis, tryptophan =  > rebeccamycin” (ID: M00789), “Aurachin biosynthesis, anthranilate =  > aurachin A” (ID: M00848), and “Pyrrolnitrin biosynthesis, tryptophan =  > pyrrolnitrin” (ID: M00790). The secondary metabolite Rebeccamycin is recognized for its anticancer activity^42^, while, Pyrrolnitrin and Aurachin are known for their antimicrobial activity^43,44^. The identification of these KEGG modules related to antimicrobial secondary metabolite illustrates the microbiome potential in both samples for secondary metabolite biosynthesis particularly, for the biosynthesis of antibiotics.

### Predictive identification of putative novel BGCs for secondary metabolites

Among the AntiSMASH-predicted BGCs, none of the NRPSs, or PKSs found a match with BGCs in the MIBiG repository that have experimentally characterized products. Further analysis of the domains of these BGCs using the NaPDoS2 web server revealed that all the ‘KS’ and ‘C’ domains of NRPS and PKS, respectively, had a percent identity of less than 85 with the existing domains in the database. According to NaPDoS2, if the domain sequence hits below 85%, it suggests that the specific domain of interest may potentially play a role in the production of uncharacterized (novel) natural products^45^. Although the percent identity for all the ‘C’ domains is significantly less than 85%, the phylogenetic tree constructed by maximum likelihood for the ‘C’ domain indicates a closeness with natural products such as pyridomycin, pristinamycin, arthrofactin, calcium-dependent antibiotics, syringomycin, and mycosubtilin with a much lower percent identity. Similarly, the phylogenetic tree constructed by maximum likelihood for the ‘KS’ domain indicates a closeness with natural products such as hedamycin, Aliivibrio fischen aryl polyene, and rishirlide. As illustrated in the phylogenetic tree, all the compounds in close proximity to the ‘C’ and ‘KS’ domains of NRPS and PKS BGCs exhibit antibiotic properties. Interestingly, this potentially indicates that the predicted novel putative BGCs might code for the machinery needed for the production of novel antibiotics. This can be supported by previous studies that identified the PKS and NRPS BGCs to be potential sources of secondary metabolites specifically antibiotics^46^. Polyketides and nonribosomal peptides are diverse classes of natural compounds that are widely recognized for their antimicrobial activities. The genetic information required for the assembly machinery involved in producing these complex compound classes is stored in the PKS and NRPS BGCs for polyketides and nonribosomal peptide compounds, respectively. The presence of novel putative PKS and NRPS BGCs in the metagenomic dataset of this study provides valuable insight into the microbial potential of these samples to produce novel secondary metabolites, most likely antibiotics.

Among the AntiSMASH-identified BGC types, two of the Terpene BGCs out of seven from sample BNFC, and one of the Ripp BGC out of two, found a match with BGCs in the MIBiG repository that have experimentally characterized natural products. The Terpene BGCs from sample BNFC were associated with Carotenoid and Hopene while the Ripp BGC from sample BNFW was associated with Mildiomycin. Further analysis of both the Terpene and Ripp BGCs involved performing a similarity search against the NCBI Metagenomics proteins (env nr) database using the BLASTn algorithm. This analysis revealed that one of the Ripp BGCs, which was associated with the natural product Mildiomycin in the MIBiG repository, aligned with the protein coenzyme PQQ synthesis protein D. On the other hand, the other Ripp BGC didn’t have a match in the repository and aligned with a hypothetical protein. Similarly, the BLASTn similarity search for the Terpene BGCs revealed that two of the Terpene BGCs, which were associated with the natural products Carotenoid and Hopene in the MIBiG repository, aligned with the proteins Lycopene cyclase and squalene hopene cyclase respectively. Lycopene cyclase is an essential enzyme involved in the biosynthesis of the natural product carotenoid. It catalyzes the cyclization of lycopene into various cyclic carotenoids. Certain types of Carotenoids are identified to possess antimicrobial properties. Squalene–hopene cyclase is an enzyme involved in the crucial steps in the biosynthesis of the natural product hopene. It catalyzes the conversion of squalene into hopene. Out of the remaining Terpene BGCs that did not find a match with BGCs in the MIBIG repository, a similarity search against the NCBI Metagenomics proteins (env nr) database revealed three terpene BGCs aligned with hypothetical proteins, one BGC aligned with unannotated protein, and one BGC aligned with uncharacterized protein. Terpene BGCs were found to dominate sample BNFC. This dominance of Terpene BGCs in soil environments is supported by other studies ^7^. Although most secondary metabolites with antimicrobial activities are produced by the assembly machinery of the PKS and NRPS BGCs^47,48^, Terpene BGCs from various life forms are widely known for their biotechnological application^49^.

Overall, the discovery of novel putative PKS and NRPS BGCs coupled with the abundant occurrence of the Terpene BGCs in sample BNFC, and the identification of the Ripp BGC in sample BNFW, unambiguously highlights the profound functional potential contained within the microbiomes, particularly in sample BNFC, for secondary metabolite biosynthesis including novel antibiotics.

## Conclusions

This study effectively achieved its aim of gaining a general understanding of the taxonomic diversity, functional potential, and biosynthetic capacity of the microbiomes from two distinctly selected natural agricultural farmlands in Ethiopia. Additionally, it successfully provided insight into the BGCs responsible for the biosynthesis of secondary metabolites. The taxonomic composition analysis results indicate the dominance of the phyla proteobacteria and actinobacteria, which are well-recognized phyla for the production of secondary metabolites including antibiotics. Furthermore, the analysis results revealed the presence of unassigned bacteria in both samples. This finding strongly suggests the availability of uncharacterized bacteria with the potential to produce novel secondary metabolites. Future investigations might include the isolation and characterization of these unassigned bacteria to exploit their untapped potential for the production of novel secondary metabolites. The functional potential analysis results unveiled the occurrence of protein domains, families, and pathways that are associated with the biosynthesis of secondary metabolites. The analysis results also revealed the presence of novel putative BGCs, such as polyketide synthases (PKS), and non-ribosomal peptide synthetase (NRPS), which might code for novel secondary metabolites, such as antibiotics. Future research endeavors might involve the expression and functional validation of these novel putative BGCs for the production of secondary metabolites. Overall, this study concludes that the microbiomes in the studied sampling locations have tremendous potential for discovering novel secondary metabolites such as antibiotics.

## Methods

### Description of the study area

**Figure 12:**
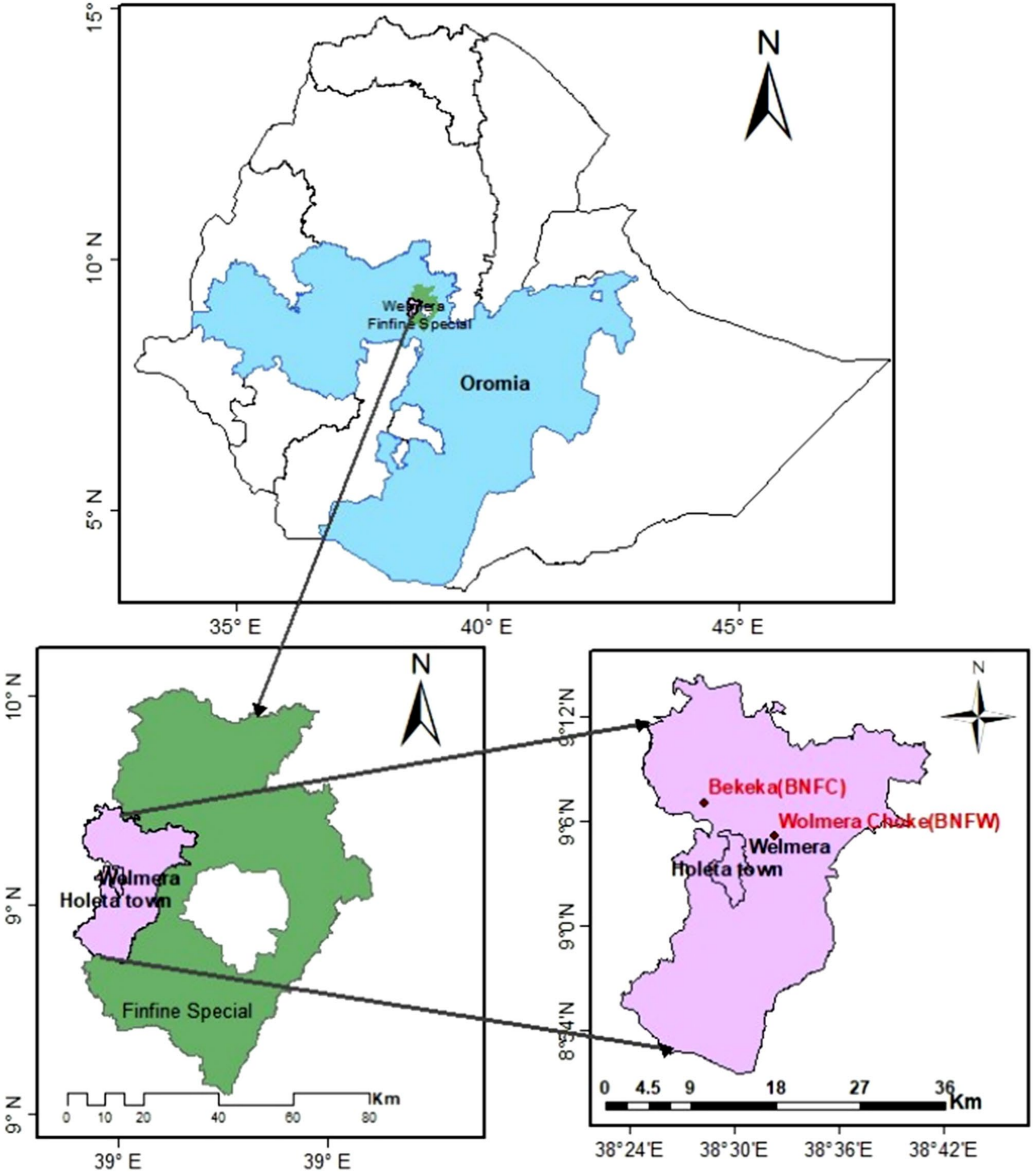
Map of sample collection sites. Bekeka Kebelle, the site from which the soil sample codded as BNFC was collected is located approximately 7 km north of Holeta town and 50 km away from Addis Ababa. Welmera Choke Kebelle from which the soil sample codded as BNFW collected is approximately 6 km east of Holeta town and 48 km away from Addis Ababa.

Two distinct natural agricultural farmlands were selected as sample collection sites for the study. The first sampling site was situated in Bekeka Kebelle, which is approximately 7 km north of Holeta town and 50 km away from Addis Ababa (Fig. 12). The kebelle is located at GPS coordinates of 9° 7′ 3.106″ N 38° 28′ 19.458″ E. The second sampling site was located in Welmera Choke Kebelle at GPS coordinates of 9° 05′ 40.6″ N 38° 32′ 20.0″ E, which is approximately 6 km east of Holeta town and 48 km away from Addis Ababa (Fig. 12). Both agricultural farmlands are privately owned and operated by farmers affiliated with the Institute for Sustainable Development (ISD), an esteemed organization dedicated to promoting sustainable agricultural practices and improving soil health in Ethiopia. The selected sampling sites have been utilized without the application of fire or synthetic chemicals and were not affected by flooding hazards. The farmland terrain at each site was characterized by a rectangular shape and zero slope, which minimized the potential confounding effects of topography on soil health and microbial diversity. The soil sample coded as BNFC was obtained from the naturally cultivated agricultural farmland located in Bekeka Kebelle (Fig. 12), at the time of collection, the farmland was covered with corn plants, and the soil had previously undergone a crop rotation cycle of animal feed, teff, and potato annually. The soil sample coded as BNFW was collected from the naturally cultivated agricultural farmland located in Welmera Choke Kebelle (Fig. 12). At the time of collection, the farmland was covered with wheat crops, and the soil had undergone an annual crop rotation cycle of onion, potato, and teff.

### Soil sample collection

The sampling protocol for this study involved the collection of soil samples from selected natural agricultural farmlands, specifically targeting both the rhizosphere and bulk soils. To ensure representative sampling, for sample BNFC, two rhizosphere soil samples were collected from the soil beneath well-grown plants at GPS coordinates of 9° 7′ 3.102″ N 38° 28′ 19.458″ E and 9° 7′ 2.634″ N 38° 28′ 19.554″ E. In addition, three bulk soil samples were obtained randomly using a 15 cm cone cutter at GPS coordinates of 9° 7′ 01.8″ N 38° 28′ 20.0″ E, 9° 07′ 02.7″ N 38° 28′ 20.7″ E, and 9° 7′ 01.8″ N 38° 28′ 20.9″E. The collected soil samples from each sampling point were subsequently, mixed to prepare representative composite samples. For the BNFW sample, a similar approach was used and two rhizospheric soil samples were collected from GPS coordinate points of 9° 05′ 39.6″ N 38° 32′ 20.0″ E and 9° 05′ 39.4″ N 38° 32′ 18.1″E. In addition, three bulk soil samples were collected randomly using a 15 cm cone cutter from GPS coordinates points of 9° 05′ 38.8" N 38° 32′ 20.4" E, 9° 05′ 38.8″ N 38° 32′ 20.6″ E, and 9° 05′ 38.8″ N 38° 32′ 20.8″E. Consequently, the collected soil samples were thoroughly mixed, homogenized, and stored in sterile polyethylene plastic bags and transported to the laboratory within an ice box for subsequent analysis. General information about the soil samples is given below in Table 6.

**Table 6:** Soil sampling information for corn and wheat Rhizosphere and Bulk soil.

Natural agricultural farmland	Sample code	Rhizosphere soil		Bulk soil	
		No. of soil samples	GPS coordinates	No. of soil samples	GPS coordinates
Corn	BNFC	2	9° 7′ 3.102″ N 38° 28′ 19.458″ E and	3	9° 7′ 01.8″ N 38° 28′ 20.0″ E,
					9° 07′ 02.7″ N 38° 28′ 20.7″ E, and
			9° 7′ 2.634″ N 38° 28′ 19.554″ E		
					• 9° 7′ 01.8″ N 38° 28′ 20.9″E
Wheat	BNFW	2	9° 05′ 39.6″ N 38° 32′ 20.0″ E and	3	9° 05′ 38.8″ N 38° 32′ 20.4″ E,
			9° 05′ 39.4″ N 38° 32′ 18.1″E		
					9° 05′ 38.8″ N 38° 32′ 20.6″ E, and
					9° 05′ 38.8″ N 38° 32′ 20.8″E

### Physicochemical analysis of soil sample

Physicochemical analyses were conducted for both samples, encompassing various standard measurements, including electrical conductivity (EC), total dissolved solids (TDS), temperature, and pH, by the utilization of Hanna HI98194 Multiparameter Portable Meter. The organic carbon content was analyzed by the Walkley–Black method with the TITREX 2000 instrument^50^. The total nitrogen content of the samples was measured using the Kjeldahal digestion method with the application of the Gerhardt KT 40 s apparatus^51^. Hydro Meter (H152) was used to measure the percentages of sand, clay, and silt in the samples^52^. The textural class of each soil sample was determined using the USDA soil triangle, and the particle density was measured using a Pycnometer^53^.

### Metagenomic DNA extraction

The metagenomic DNA was extracted with slight modifications from the extraction protocol used by Verma 2017^54^, making sure the protocol meets our specific experimental conditions. Briefly, five grams of soil samples were taken from each of the two soil types and mixed with 10 ml of extraction buffer [containing 100 mM Tris/HCl (pH 8.0), 100 mM EDTA (pH 8.0), 100 mM sodium phosphate buffer (pH 8.0), 1.5 M NaCl, 1% (w/v) CTAB, and 100 mM CaCl2]. Pestle and mortar were utilized to mix thoroughly the soil sample and the extraction buffer and the mixture was transferred into a screw cap tube. Subsequently, 100 µL of proteinase, 100 µL of lysozyme, and 2 mL of 20% (w/v) SDS were added to the mixture. The resulting mixture was vortexed for a duration of 4–6 min. After vortexing, the mixture was centrifuged at 3500 × for 30 min, and the first supernatant was collected. Two (2) mL of x% of SDS and 10 mL of extraction buffer were used for treating the remaining pellet. To obtain a second supernatant the mixture was vortexed and centrifuged. The two supernatants were combined by mixing them. To the combined supernatants an equal volume of chloroform isoamyl alcohol was added and the resulting mixture was vortexed and centrifuged at 3500 xg for 30 min. After centrifugation, the aqueous phase was collected. Subsequently, 1 ml of the aqueous phase was mixed with 0.7 ml of isopropanol which is ice-cold, and was incubated for 2 h at − 20 °C. From the mixture, seven hundred microliters (700 µL) were poured into the mini spin column (EXgene™), and the column was centrifuged for 30 s at 1200 g. The process of adding 700 µL of the mixture to the mini spin column was repeated until the entire mixture was utilized. The extracted DNA was washed by adding 700 µL of 5 M NaCl to the mini spin column and centrifuging it for 30 s. For further purification, the mini spin column was treated with 500 µL of 70% ethanol and centrifuged for 30 s. Finally, the DNA attached to the membrane of the mini-spin column was eluted by adding 70 µL of Tris EDTA buffer by centrifuging it for one minute.

### Quantification of the metagenomic DNA

The extracted metagenomic DNA concentration and purity were evaluated by Thermo Scientific NANODROP 2000 instrument, and the integrity of the DNA was assessed by 0.8% agarose gel electrophoresis, 2 μl of gel red, and 2 μl of loading dye was mixed with 3 μl of the DNA sample before loading. UVITEC gel documentation system was used for visualization.

### Library preparation and sequencing

The extracted metagenomic DNA was sent to the Singapore Novogene company for shotgun sequencing. Illumina NovaSeq 6000 sequencing platform was utilized for sequencing. To construct the library, the genomic DNA was randomly fragmented into shorter pieces, followed by end repair, A-tailing, and ligation with Illumina adapters. The resulting fragments, along with the adapters, underwent PCR amplification, size selection, and purification. Subsequently, the library was subjected to quantification using Qubit and real-time PCR, while size distribution detection was performed using a bioanalyzer. Based on effective library concentration and the amount of data needed the libraries were measured and pooled. Consequently, the pooled libraries were sequenced using Illumina sequencing platforms.

### Raw reads quality control

The raw reads obtained from sequencing were uploaded to the Galaxy Australia server^55^ for quality control. The initial stage of quality control involved the removal of the plant and human host genome. The meticulous execution of removing the plant and human host genomes from the raw reads was accomplished by mapping the raw reads to the host genomes using the BWA-MEM Galaxy tool version 0.7.17.2. Then after, a comprehensive evaluation of the raw read quality was conducted using FASTQC Galaxy version 0.73. The high-quality metagenome sequences were used for genome assembly.

### Metagenome assembly

The metagenomic data set underwent de novo assembly utilizing MEGAHIT Galaxy version 1.2.9 and metaSPAdes Galaxy version 3.15.4. The output assemblies from each of the assemblers were compared using Quast (Quality Assessment Tool for Genome Assemblies) Galaxy version 5.2.0. Then the assembled genome were used for taxonomic annotation and gene prediction.

### Taxonomic annotation

The assembled contigs were uploaded to the Galaxy Europe server^56^ The cmsearch tool, Galaxy version 1.1.4, was utilized to search the query sequence against the locally installed covariance model of RFAM 11.0, dated 2.09.2013. Subsequently, the Easel tool version 0.48 was employed to extract small subunit (SSU) and large subunit (LSU) rRNA coordinates and sequences from the output. The sequences were taxonomically classified using the MapSeq tool^57^, which mapped them against both hierarchically clustered and annotated reference sequences. The results from MapSeq were then visualized using Krona Galaxy version 2.6.1.1 and the web-based plotly chart studio^58^

### Gene prediction and functional annotation

FragGeneScan Galaxy version 1.30.0 was employed on the Galaxy Europe server to identify protein-coding DNA sequences (pCDS) from the processed contigs. The predicted genes were subsequently subjected to functional annotation by querying them against the InterPro database using the InterProScan tool, Galaxy version 5.59–91.0. The results from the InterPro database, Pfam database, and Gene Ontology (GO) were extracted from the output. This extraction process was conducted on Ubuntu, utilizing the command-line tool 'grep'. A summary file was generated for each database's outputs, which included the assigned number of protein-coding DNA sequences, the database accession ID, and a description. This extraction process was performed using the command-line utility ‘cut’. Lastly, the top ten highest results from the three databases were extracted using the ‘sort’ command-line tool and were visualized using the Plotly chart studio.

### KEGG orthologs and pathway analysis

The KEGG Automatic Annotation Server (KAAS) website^59^ was utilized to access the KEGG database^60^ for KEGG orthologs (KOs) annotation and KEGG module analysis. The translated gene sequences from FragGeneScan were uploaded to the KAAS server, and the query sequences were annotated using the GHOSTX search and SBH method. The results were visualized using the plotly chart studio.

### Biosynthetic gene clusters (BGCs) prediction

AntiSMASH bioinformatics Galaxy version 6.1.1, was employed to predict the BGCs from the assembled contigs. The predicted BGCs from the dataset were compared with entries in the MIBiGo repository to identify BGCs without a match. Those BGCs without a match were selected for subsequent analysis.

### Identification of putative novel BGCs for natural product

The polyketide synthase (PKS) and non-ribosomal peptide synthetase (NRPS) biosynthetic gene clusters (BGCs), which were predicted by antiSMASH and have no match with the MIBiG repository, were further analyzed to determine their novelty. This was done by querying them using the tool NaPDoS2^61^ the second-generation Natural Product Domain Seeker, to analyze the ketosynthase (KS) domain from PKS and the condensation (C) domains from NRPS. A phylogeny-based classification scheme was utilized to make broader predictions about the PKS and NRPS genes in which these domains were identified. This approach included specific product classification assignments. The terpene and Ripp BGCs were further analyzed to determine their novelty by utilizing the BLASTn algorithm. This involved comparing the BGCs against the NCBI Metagenomics proteins (env nr) database.

### Supplementary Information

Supplementary Figure 1.

Supplementary Figure 2.

Supplementary Figure 3.

Supplementary Figure 4.

Supplementary Table 1.

Supplementary Table 2.

Supplementary Table 3.

Supplementary Table 4.

Supplementary Table 5.

Supplementary Table 6.

## Data Availability

The shotgun raw reads sequence data was uploaded to the European Nucleotide Archive (ENA) and is available under the project accession PRJEB58067. The Sequence accession for sample BNFC is ERR10638405 and for sample BNFW is ERR106295.According to ENA data policy, t he data can only be accessed after publication. For review purpose, the data can accessed by using our webin submission account and password provided to us by ENA. This is available https://www.ebi.ac.uk/ena/submit/webin/login. Webin submission account: Webin-63572. Password: *Varune1238. The raw reads are located in “Run Files Report” under “Raw Reads (Experiments and Runs)”.

## Abbreviations

ABC-transporter: ATP-Binding Cassette transporter
AD: Acyl-ACP Dehydrogenase domain
AMR: Antimicrobial Resistance
AntiSMASH: Antibiotics & Secondary Metabolite Analysis Shell
BNFC: Soil sample collected from Bekeka Kebelle
BNFW: Soil sample collected from Welmera Choke Kebelle
BGC: Biosynthetic Gene Cluster
BLASTn: Basic Local Alignment Search Tool nucleotide
C: Condensation domain
CDS: Coding DNA Sequence
CTAB: Cetyltrimethylammonium Bromide
EC: Electrical conductivity
ECF: Extra Cytoplasmic Function
EDTA: Ethylenediaminetetraacetic Acid
ENA: European Nucleotide Archive
eDNA: Environmental DNA
FACS-based: Fluorescence-Activated Cell Sorting-based
GO: Gene Ontology
GPS: Global Positioning System
ISD: Institute of sustainable development
KAAS: KEGG Automatic Annotation Server
KEGG: Kyoto Encyclopedia of Genes and Genomes
KO: KEGG Orthology
KS: Ketosynthase domain
MIBiG: Minimum Information about a Biosynthetic Gene cluster
NaPDoS2: Natural Product Domain Seeker 2
NCBI: National Center for Biotechnology Information
NPs: Natural products
NRPS: Nonribosomal Peptide Synthetase
pCDS: Predicted Coding DNA Sequence
Ripp: Ribosomally synthesized and post-translationally modified peptides
SDS: Sodium Dodecyl Sulfate
SSU: Small Subunit (of ribosome)
TBDR: TonB-dependent receptor
TDS: Total dissolved solids
