# Supplementary material for: Shotgun metagenomic insights into secondary metabolite biosynthetic gene clusters reveal taxonomic and functional profiles of microbiomes in natural farmland soil
Source: Sci Rep. 2024 Jul 2;14:15096. doi: 10.1038/s41598-024-63254-x (PMC11220033; doi:10.1038/s41598-024-63254-x)
Supplement: Supplementary file 1 — Supplementary Figure 1. [file 41598_2024_63254_MOESM1_ESM.docx]

**Supplementary Figure 1** The InterPro entry summary for sample BNFW indicates that the "Winged helix-like DNA-binding domain superfamily" had the highest number of matched CDSs, with the "Alpha/Beta hydrolase fold" entry coming in second.
