# Supplementary material for: Shotgun metagenomic insights into secondary metabolite biosynthetic gene clusters reveal taxonomic and functional profiles of microbiomes in natural farmland soil
Source: Sci Rep. 2024 Jul 2;14:15096. doi: 10.1038/s41598-024-63254-x (PMC11220033; doi:10.1038/s41598-024-63254-x)
Supplement: Supplementary file 2 — Supplementary Figure 2. [file 41598_2024_63254_MOESM2_ESM.docx]

**Supplementary Figure 2** Here are the top ten Pfam entries for sample BNFW, along with their respective IDs, descriptions, and total pCDS counts. The entries with the highest number of pCDSs assigned are the "ABC transporter," "Response regulator receiver domain," and "Binding-protein-dependent transport system inner membrane component," respectively.
