# Supplementary material for: Shotgun metagenomic insights into secondary metabolite biosynthetic gene clusters reveal taxonomic and functional profiles of microbiomes in natural farmland soil
Source: Sci Rep. 2024 Jul 2;14:15096. doi: 10.1038/s41598-024-63254-x (PMC11220033; doi:10.1038/s41598-024-63254-x)
Supplement: Supplementary file 3 — Supplementary Figure 3. [file 41598_2024_63254_MOESM3_ESM.docx]

**Supplementary Figure 3** Summary of GO term annotations for sample BNFW: There were 33,171 annotations for Biological Process (A), 8,367 for Cellular Component (B), and 45,586 for Molecular Function (C).
