# Supplementary material for: Shotgun metagenomic insights into secondary metabolite biosynthetic gene clusters reveal taxonomic and functional profiles of microbiomes in natural farmland soil
Source: Sci Rep. 2024 Jul 2;14:15096. doi: 10.1038/s41598-024-63254-x (PMC11220033; doi:10.1038/s41598-024-63254-x)
Supplement: Supplementary file 4 — Supplementary Figure 4. [file 41598_2024_63254_MOESM4_ESM.docx]

**Supplementary Figure 4** Here are the top ten KO entries for sample BNFW, including their class IDs, descriptions, and the number of pCDSs assigned to each. The "RNA polymerase sigma-70 factor, ECF subfamily," "putative ABC transport system permease protein," and "Transposase" are the top three KO classes with the highest number of assigned pCDSs, respectively.
