# Supplementary material for: Shotgun metagenomic insights into secondary metabolite biosynthetic gene clusters reveal taxonomic and functional profiles of microbiomes in natural farmland soil
Source: Sci Rep. 2024 Jul 2;14:15096. doi: 10.1038/s41598-024-63254-x (PMC11220033; doi:10.1038/s41598-024-63254-x)
Supplement: Supplementary file 5 — Supplementary Table 1. [file 41598_2024_63254_MOESM5_ESM.docx]

Supplementary Table 1. KEGG module annotation for sample BNFC

| module_accession | completeness | pathway_name | pathway_class |
| --- | --- | --- | --- |
| M00001 | 100 | Glycolysis (Embden-Meyerhof pathway), glucose => pyruvate | Pathway modules; Carbohydrate metabolism; Central carbohydrate metabolism |
| M00002 | 100 | Glycolysis, core module involving three-carbon compounds | Pathway modules; Carbohydrate metabolism; Central carbohydrate metabolism |
| M00003 | 100 | Gluconeogenesis, oxaloacetate => fructose-6P | Pathway modules; Carbohydrate metabolism; Central carbohydrate metabolism |
| M00004 | 100 | Pentose phosphate pathway (Pentose phosphate cycle) | Pathway modules; Carbohydrate metabolism; Central carbohydrate metabolism |
| M00005 | 100 | PRPP biosynthesis, ribose 5P => PRPP | Pathway modules; Carbohydrate metabolism; Central carbohydrate metabolism |
| M00006 | 100 | Pentose phosphate pathway, oxidative phase, glucose 6P => ribulose 5P | Pathway modules; Carbohydrate metabolism; Central carbohydrate metabolism |
| M00007 | 100 | Pentose phosphate pathway, non-oxidative phase, fructose 6P => ribose 5P | Pathway modules; Carbohydrate metabolism; Central carbohydrate metabolism |
| M00020 | 100 | Serine biosynthesis, glycerate-3P => serine | Pathway modules; Amino acid metabolism; Serine and threonine metabolism |
| M00021 | 100 | Cysteine biosynthesis, serine => cysteine | Pathway modules; Amino acid metabolism; Cysteine and methionine metabolism |
| M00022 | 100 | Shikimate pathway, phosphoenolpyruvate + erythrose-4P => chorismate | Pathway modules; Amino acid metabolism; Aromatic amino acid metabolism |
| M00023 | 100 | Tryptophan biosynthesis, chorismate => tryptophan | Pathway modules; Amino acid metabolism; Aromatic amino acid metabolism |
| M00028 | 100 | Ornithine biosynthesis, glutamate => ornithine | Pathway modules; Amino acid metabolism; Arginine and proline metabolism |
| M00031 | 100 | Lysine biosynthesis, mediated by LysW, 2-aminoadipate => lysine | Pathway modules; Amino acid metabolism; Lysine metabolism |
| M00049 | 100 | Adenine ribonucleotide biosynthesis, IMP => ADP,ATP | Pathway modules; Nucleotide metabolism; Purine metabolism |
| M00050 | 100 | Guanine ribonucleotide biosynthesis IMP => GDP,GTP | Pathway modules; Nucleotide metabolism; Purine metabolism |
| M00052 | 100 | Pyrimidine ribonucleotide biosynthesis, UMP => UDP/UTP,CDP/CTP | Pathway modules; Nucleotide metabolism; Pyrimidine metabolism |
| M00053 | 100 | Pyrimidine deoxyribonuleotide biosynthesis, CDP/CTP => dCDP/dCTP,dTDP/dTTP | Pathway modules; Nucleotide metabolism; Pyrimidine metabolism |
| M00063 | 100 | CMP-KDO biosynthesis | Pathway modules; Glycan metabolism; Lipopolysaccharide metabolism |
| M00083 | 100 | Fatty acid biosynthesis, elongation | Pathway modules; Lipid metabolism; Fatty acid metabolism |
| M00086 | 100 | beta-Oxidation, acyl-CoA synthesis | Pathway modules; Lipid metabolism; Fatty acid metabolism |
| M00088 | 100 | Ketone body biosynthesis, acetyl-CoA => acetoacetate/3-hydroxybutyrate/acetone | Pathway modules; Lipid metabolism; Lipid metabolism |
| M00091 | 100 | Phosphatidylcholine (PC) biosynthesis, PE => PC | Pathway modules; Lipid metabolism; Lipid metabolism |
| M00093 | 100 | Phosphatidylethanolamine (PE) biosynthesis, PA => PS => PE | Pathway modules; Lipid metabolism; Lipid metabolism |
| **M00096** | **100** | **C5 isoprenoid biosynthesis, non-mevalonate pathway** | **Pathway modules; Biosynthesis of terpenoids and polyketides; Terpenoid backbone biosynthesis** |
| M00115 | 100 | NAD biosynthesis, aspartate => NAD | Pathway modules; Metabolism of cofactors and vitamins; Cofactor and vitamin metabolism |
| M00118 | 100 | Glutathione biosynthesis, glutamate => glutathione | Pathway modules; Amino acid metabolism; Other amino acid metabolism |
| M00119 | 100 | Pantothenate biosynthesis, valine/L-aspartate => pantothenate | Pathway modules; Metabolism of cofactors and vitamins; Cofactor and vitamin metabolism |
| M00120 | 100 | Coenzyme A biosynthesis, pantothenate => CoA | Pathway modules; Metabolism of cofactors and vitamins; Cofactor and vitamin metabolism |
| M00121 | 100 | Heme biosynthesis, plants and bacteria, glutamate => heme | Pathway modules; Metabolism of cofactors and vitamins; Cofactor and vitamin metabolism |
| M00122 | 100 | Cobalamin biosynthesis, cobinamide => cobalamin | Pathway modules; Metabolism of cofactors and vitamins; Cofactor and vitamin metabolism |
| M00125 | 100 | Riboflavin biosynthesis, GTP => riboflavin/FMN/FAD | Pathway modules; Metabolism of cofactors and vitamins; Cofactor and vitamin metabolism |
| M00126 | 100 | Tetrahydrofolate biosynthesis, GTP => THF | Pathway modules; Metabolism of cofactors and vitamins; Cofactor and vitamin metabolism |
| M00127 | 100 | Thiamine biosynthesis, AIR => thiamine-P/thiamine-2P | Pathway modules; Metabolism of cofactors and vitamins; Cofactor and vitamin metabolism |
| M00133 | 100 | Polyamine biosynthesis, arginine => agmatine => putrescine => spermidine | Pathway modules; Amino acid metabolism; Polyamine biosynthesis |
| M00134 | 100 | Polyamine biosynthesis, arginine => ornithine => putrescine | Pathway modules; Amino acid metabolism; Polyamine biosynthesis |
| M00140 | 100 | C1-unit interconversion, prokaryotes | Pathway modules; Metabolism of cofactors and vitamins; Cofactor and vitamin metabolism |
| M00151 | 100 | Cytochrome bc1 complex respiratory unit | Pathway modules; Energy metabolism; ATP synthesis |
| M00157 | 100 | F-type ATPase, prokaryotes and chloroplasts | Pathway modules; Energy metabolism; ATP synthesis |
| M00169 | 100 | CAM (Crassulacean acid metabolism), light | Pathway modules; Energy metabolism; Carbon fixation |
| M00307 | 100 | Pyruvate oxidation, pyruvate => acetyl-CoA | Pathway modules; Carbohydrate metabolism; Central carbohydrate metabolism |
| **M00364** | **100** | **C10-C20 isoprenoid biosynthesis, bacteria** | **Pathway modules; Biosynthesis of terpenoids and polyketides; Terpenoid backbone biosynthesis** |
| M00378 | 100 | F420 biosynthesis | Pathway modules; Energy metabolism; Methane metabolism |
| M00432 | 100 | Leucine biosynthesis, 2-oxoisovalerate => 2-oxoisocaproate | Pathway modules; Amino acid metabolism; Branched-chain amino acid metabolism |
| M00530 | 100 | Dissimilatory nitrate reduction, nitrate => ammonia | Pathway modules; Energy metabolism; Nitrogen metabolism |
| M00532 | 100 | Photorespiration | Pathway modules; Carbohydrate metabolism; Other carbohydrate metabolism |
| M00579 | 100 | Phosphate acetyltransferase-acetate kinase pathway, acetyl-CoA => acetate | Pathway modules; Energy metabolism; Carbon fixation |
| M00763 | 100 | Ornithine biosynthesis, mediated by LysW, glutamate => ornithine | Pathway modules; Amino acid metabolism; Arginine and proline metabolism |
| **M00793** | **100** | **dTDP-L-rhamnose biosynthesis** | **Pathway modules; Biosynthesis of terpenoids and polyketides; Polyketide sugar unit biosynthesis** |
| M00844 | 100 | Arginine biosynthesis, ornithine => arginine | Pathway modules; Amino acid metabolism; Arginine and proline metabolism |
| M00846 | 100 | Siroheme biosynthesis, glutamate => siroheme | Pathway modules; Metabolism of cofactors and vitamins; Cofactor and vitamin metabolism |
| M00854 | 100 | Glycogen biosynthesis, glucose-1P => glycogen/starch | Pathway modules; Carbohydrate metabolism; Other carbohydrate metabolism |
| M00866 | 100 | KDO2-lipid A biosynthesis, Raetz pathway, non-LpxL-LpxM type | Pathway modules; Glycan metabolism; Lipopolysaccharide metabolism |
| M00868 | 100 | Heme biosynthesis, animals and fungi, glycine => heme | Pathway modules; Metabolism of cofactors and vitamins; Cofactor and vitamin metabolism |
| M00880 | 100 | Molybdenum cofactor biosynthesis, GTP => molybdenum cofactor | Pathway modules; Metabolism of cofactors and vitamins; Cofactor and vitamin metabolism |
| M00048 | 95.83 | Inosine monophosphate biosynthesis, PRPP + glutamine => IMP | Pathway modules; Nucleotide metabolism; Purine metabolism |
| **M00849** | **91.67** | **C5 isoprenoid biosynthesis, mevalonate pathway, archaea** | **Pathway modules; Biosynthesis of terpenoids and polyketides; Terpenoid backbone biosynthesis** |
| M00016 | 88.89 | Lysine biosynthesis, succinyl-DAP pathway, aspartate => lysine | Pathway modules; Amino acid metabolism; Lysine metabolism |
| M00051 | 88.89 | Uridine monophosphate biosynthesis, glutamine (+ PRPP) => UMP | Pathway modules; Nucleotide metabolism; Pyrimidine metabolism |
| M00060 | 88.89 | KDO2-lipid A biosynthesis, Raetz pathway, LpxL-LpxM type | Pathway modules; Glycan metabolism; Lipopolysaccharide metabolism |
| M00082 | 87.5 | Fatty acid biosynthesis, initiation | Pathway modules; Lipid metabolism; Fatty acid metabolism |
| **M00095** | **85.71** | **C5 isoprenoid biosynthesis, mevalonate pathway** | **Pathway modules; Biosynthesis of terpenoids and polyketides; Terpenoid backbone biosynthesis** |
| M00167 | 85.71 | Reductive pentose phosphate cycle, glyceraldehyde-3P => ribulose-5P | Pathway modules; Energy metabolism; Carbon fixation |
| M00036 | 83.33 | Leucine degradation, leucine => acetoacetate + acetyl-CoA | Pathway modules; Amino acid metabolism; Branched-chain amino acid metabolism |
| M00123 | 83.33 | Biotin biosynthesis, pimeloyl-ACP/CoA => biotin | Pathway modules; Metabolism of cofactors and vitamins; Cofactor and vitamin metabolism |
| M00156 | 83.33 | Cytochrome c oxidase, cbb3-type | Pathway modules; Energy metabolism; ATP synthesis |
| M00565 | 83.33 | Trehalose biosynthesis, D-glucose 1P => trehalose | Pathway modules; Carbohydrate metabolism; Other carbohydrate metabolism |
| M00572 | 83.33 | Pimeloyl-ACP biosynthesis, BioC-BioH pathway, malonyl-ACP => pimeloyl-ACP | Pathway modules; Metabolism of cofactors and vitamins; Cofactor and vitamin metabolism |
| M00018 | 80 | Threonine biosynthesis, aspartate => homoserine => threonine | Pathway modules; Amino acid metabolism; Serine and threonine metabolism |
| M00029 | 80 | Urea cycle | Pathway modules; Amino acid metabolism; Arginine and proline metabolism |
| M00044 | 80 | Tyrosine degradation, tyrosine => homogentisate | Pathway modules; Amino acid metabolism; Aromatic amino acid metabolism |
| M00061 | 80 | D-Glucuronate degradation, D-glucuronate => pyruvate + D-glyceraldehyde 3P | Pathway modules; Carbohydrate metabolism; Other carbohydrate metabolism |
| M00552 | 80 | D-galactonate degradation, De Ley-Doudoroff pathway, D-galactonate => glycerate-3P | Pathway modules; Carbohydrate metabolism; Other carbohydrate metabolism |
| M00570 | 80 | Isoleucine biosynthesis, threonine => 2-oxobutanoate => isoleucine | Pathway modules; Amino acid metabolism; Branched-chain amino acid metabolism |
