# Supplementary material for: Shotgun metagenomic insights into secondary metabolite biosynthetic gene clusters reveal taxonomic and functional profiles of microbiomes in natural farmland soil
Source: Sci Rep. 2024 Jul 2;14:15096. doi: 10.1038/s41598-024-63254-x (PMC11220033; doi:10.1038/s41598-024-63254-x)
Supplement: Supplementary file 7 — Supplementary Table 3. [file 41598_2024_63254_MOESM7_ESM.docx]

Supplementary Table 3. Taxonomic assignment results for sample BNFC

| Taxonomy |
| --- |
| sk__Archaea;k__;p__Thaumarchaeota;c__Nitrososphaeria;o__Nitrososphaerales;f__Nitrososphaeraceae |
| sk__Bacteria |
| sk__Bacteria;k__;p__Acidobacteria |
| sk__Bacteria;k__;p__Acidobacteria;c__Acidobacteriia;o__Bryobacterales;f__Solibacteraceae;g__Candidatus_Solibacter |
| sk__Bacteria;k__;p__Acidobacteria;c__Blastocatellia;o__Blastocatellales;f__Blastocatellaceae |
| sk__Bacteria;k__;p__Acidobacteria;c__Blastocatellia;o__Blastocatellales;f__Pyrinomonadaceae |
| sk__Bacteria;k__;p__Acidobacteria;c__Holophagae |
| sk__Bacteria;k__;p__Actinobacteria;c__Acidimicrobiia |
| sk__Bacteria;k__;p__Actinobacteria;c__Actinobacteria |
| sk__Bacteria;k__;p__Actinobacteria;c__Actinobacteria;o__Micrococcales |
| sk__Bacteria;k__;p__Actinobacteria;c__Actinobacteria;o__Micrococcales;f__Micrococcaceae |
| sk__Bacteria;k__;p__Actinobacteria;c__Actinobacteria;o__Propionibacteriales;f__Nocardioidaceae;g__Marmoricola |
| sk__Bacteria;k__;p__Actinobacteria;c__Thermoleophilia;o__Solirubrobacterales |
| sk__Bacteria;k__;p__Bacteroidetes;c__Chitinophagia;o__Chitinophagales;f__Chitinophagaceae |
| sk__Bacteria;k__;p__Bacteroidetes;c__Sphingobacteriia;o__Sphingobacteriales;f__Sphingobacteriaceae |
| sk__Bacteria;k__;p__Candidatus_Kuenenbacteria |
| sk__Bacteria;k__;p__Candidatus_Rokubacteria;c__;o__;f__;g__;s__Candidatus_Rokubacteria_bacterium_CSP1-6 |
| sk__Bacteria;k__;p__Candidatus_Saccharibacteria |
| sk__Bacteria;k__;p__Chloroflexi |
| sk__Bacteria;k__;p__Chloroflexi;c__Anaerolineae |
| sk__Bacteria;k__;p__Chloroflexi;c__Anaerolineae;o__Anaerolineales;f__Anaerolineaceae |
| sk__Bacteria;k__;p__Chloroflexi;c__Chloroflexia;o__Chloroflexales;f__Roseiflexaceae |
| sk__Bacteria;k__;p__Gemmatimonadetes;c__Gemmatimonadetes;o__Gemmatimonadales;f__Gemmatimonadaceae |
| sk__Bacteria;k__;p__Nitrospirae;c__Nitrospira |
| sk__Bacteria;k__;p__Proteobacteria;c__Alphaproteobacteria;o__Holosporales;f__Caedimonadaceae;g__Candidatus_Nucleicultrix |
| sk__Bacteria;k__;p__Proteobacteria;c__Alphaproteobacteria;o__Rhizobiales;f__Hyphomicrobiaceae;g__Rhodoplanes |
| sk__Bacteria;k__;p__Proteobacteria;c__Alphaproteobacteria;o__Rhodospirillales;f__;g__Reyranella |
| sk__Bacteria;k__;p__Proteobacteria;c__Alphaproteobacteria;o__Sphingomonadales;f__Sphingomonadaceae |
| sk__Bacteria;k__;p__Proteobacteria;c__Betaproteobacteria;o__Nitrosomonadales;f__Nitrosomonadaceae |
| sk__Bacteria;k__;p__Proteobacteria;c__Deltaproteobacteria |
| sk__Bacteria;k__;p__Proteobacteria;c__Deltaproteobacteria;o__Desulfarculales;f__Desulfarculaceae |
| sk__Bacteria;k__;p__Proteobacteria;c__Deltaproteobacteria;o__Myxococcales |
| sk__Bacteria;k__;p__Proteobacteria;c__Gammaproteobacteria |
| sk__Bacteria;k__;p__Proteobacteria;c__Gammaproteobacteria;o__;f__;g__Acidibacter |
| sk__Bacteria;k__;p__Proteobacteria;c__Gammaproteobacteria;o__Pseudomonadales;f__Pseudomonadaceae;g__Pseudomonas |
| sk__Bacteria;k__;p__Proteobacteria;c__Gammaproteobacteria;o__Pseudomonadales;f__Pseudomonadaceae;g__Pseudomonas;s__Pseudomonas_moraviensis |
| sk__Bacteria;k__;p__Proteobacteria;c__Gammaproteobacteria;o__Xanthomonadales;f__Xanthomonadaceae;g__Arenimonas |
| sk__Bacteria;k__;p__Verrucomicrobia;c__Spartobacteria;o__Chthoniobacterales;f__Chthoniobacteraceae |
| sk__Bacteria;k__;p__Verrucomicrobia;c__Verrucomicrobiae |
| sk__Eukaryota |
| sk__Eukaryota;k__Fungi;p__Mucoromycota |
| sk__Eukaryota;k__Viridiplantae;p__Streptophyta;c__Magnoliopsida |
| sk__Eukaryota;k__Viridiplantae;p__Streptophyta;c__Magnoliopsida;o__Poales |
