# Supplementary material for: Shotgun metagenomic insights into secondary metabolite biosynthetic gene clusters reveal taxonomic and functional profiles of microbiomes in natural farmland soil
Source: Sci Rep. 2024 Jul 2;14:15096. doi: 10.1038/s41598-024-63254-x (PMC11220033; doi:10.1038/s41598-024-63254-x)
Supplement: Supplementary file 8 — Supplementary Table 4. [file 41598_2024_63254_MOESM8_ESM.docx]

Supplementary Table 4. Taxonomic assignment results for sample BNFW

| Taxonomy |
| --- |
| sk__Archaea;k__;p__Thaumarchaeota;c__Nitrososphaeria;o__Nitrososphaerales;f__Nitrososphaeraceae |
| sk__Bacteria |
| sk__Bacteria;k__;p__Acidobacteria |
| sk__Bacteria;k__;p__Acidobacteria;c__Acidobacteriia |
| sk__Bacteria;k__;p__Acidobacteria;c__Acidobacteriia;o__Bryobacterales;f__Solibacteraceae;g__Candidatus_Solibacter |
| sk__Bacteria;k__;p__Acidobacteria;c__Blastocatellia;o__Blastocatellales;f__Pyrinomonadaceae |
| sk__Bacteria;k__;p__Actinobacteria |
| sk__Bacteria;k__;p__Actinobacteria;c__Acidimicrobiia;o__Acidimicrobiales;f__Iamiaceae;g__Iamia |
| sk__Bacteria;k__;p__Actinobacteria;c__Acidimicrobiia;o__Acidimicrobiales;f__Ilumatobacteraceae |
| sk__Bacteria;k__;p__Actinobacteria;c__Actinobacteria;o__Geodermatophilales;f__Geodermatophilaceae;g__Blastococcus |
| sk__Bacteria;k__;p__Actinobacteria;c__Actinobacteria;o__Micrococcales;f__Intrasporangiaceae;g__Tetrasphaera |
| sk__Bacteria;k__;p__Actinobacteria;c__Actinobacteria;o__Micrococcales;f__Micrococcaceae |
| sk__Bacteria;k__;p__Actinobacteria;c__Actinobacteria;o__Pseudonocardiales;f__Pseudonocardiaceae |
| sk__Bacteria;k__;p__Actinobacteria;c__Thermoleophilia;o__Solirubrobacterales |
| sk__Bacteria;k__;p__Bacteroidetes |
| sk__Bacteria;k__;p__Bacteroidetes;c__Bacteroidia;o__Bacteroidales;f__Rikenellaceae;g__Rikenella |
| sk__Bacteria;k__;p__Bacteroidetes;c__Chitinophagia;o__Chitinophagales;f__Chitinophagaceae |
| sk__Bacteria;k__;p__Bacteroidetes;c__Cytophagia;o__Cytophagales;f__Microscillaceae |
| sk__Bacteria;k__;p__Bacteroidetes;c__Sphingobacteriia;o__Sphingobacteriales |
| sk__Bacteria;k__;p__Chloroflexi |
| sk__Bacteria;k__;p__Chloroflexi;c__Anaerolineae;o__Anaerolineales;f__Anaerolineaceae |
| sk__Bacteria;k__;p__Chloroflexi;c__Chloroflexia;o__Chloroflexales |
| sk__Bacteria;k__;p__Gemmatimonadetes;c__Longimicrobia;o__Longimicrobiales;f__Longimicrobiaceae |
| sk__Bacteria;k__;p__Nitrospirae;c__Nitrospira |
| sk__Bacteria;k__;p__Planctomycetes |
| sk__Bacteria;k__;p__Planctomycetes;c__Phycisphaerae;o__Tepidisphaerales |
| sk__Bacteria;k__;p__Planctomycetes;c__Planctomycetia;o__Pirellulales;f__Pirellulaceae |
| sk__Bacteria;k__;p__Proteobacteria;c__Alphaproteobacteria |
| sk__Bacteria;k__;p__Proteobacteria;c__Alphaproteobacteria;o__Rhizobiales;f__Bradyrhizobiaceae |
| sk__Bacteria;k__;p__Proteobacteria;c__Alphaproteobacteria;o__Rhizobiales;f__Xanthobacteraceae |
| sk__Bacteria;k__;p__Proteobacteria;c__Betaproteobacteria;o__Burkholderiales |
| sk__Bacteria;k__;p__Proteobacteria;c__Betaproteobacteria;o__Nitrosomonadales;f__Nitrosomonadaceae |
| sk__Bacteria;k__;p__Proteobacteria;c__Deltaproteobacteria |
| sk__Bacteria;k__;p__Proteobacteria;c__Deltaproteobacteria;o__Desulfarculales;f__Desulfarculaceae |
| sk__Bacteria;k__;p__Proteobacteria;c__Deltaproteobacteria;o__Myxococcales |
| sk__Bacteria;k__;p__Proteobacteria;c__Deltaproteobacteria;o__Myxococcales;f__Kofleriaceae;g__Haliangium |
| sk__Bacteria;k__;p__Proteobacteria;c__Gammaproteobacteria |
| sk__Bacteria;k__;p__Proteobacteria;c__Gammaproteobacteria;o__Enterobacterales;f__Enterobacteriaceae |
| sk__Bacteria;k__;p__Proteobacteria;c__Gammaproteobacteria;o__Nevskiales;f__Steroidobacteraceae |
| sk__Bacteria;k__;p__Proteobacteria;c__Gammaproteobacteria;o__Pseudomonadales |
| sk__Bacteria;k__;p__Proteobacteria;c__Gammaproteobacteria;o__Xanthomonadales;f__Rhodanobacteraceae |
| sk__Bacteria;k__;p__Verrucomicrobia;c__Opitutae;o__Opitutales;f__Opitutaceae |
| sk__Bacteria;k__;p__Verrucomicrobia;c__Verrucomicrobiae |
| sk__Eukaryota |
| sk__Eukaryota;k__Fungi;p__Ascomycota;c__Sordariomycetes |
| sk__Eukaryota;k__Metazoa;p__Arthropoda;c__Insecta;o__Hemiptera |
| sk__Eukaryota;k__Viridiplantae;p__Streptophyta;c__Magnoliopsida |
| sk__Eukaryota;k__Viridiplantae;p__Streptophyta;c__Magnoliopsida;o__Poales;f__Poaceae |
