# Supplementary material for: Shotgun metagenomic insights into secondary metabolite biosynthetic gene clusters reveal taxonomic and functional profiles of microbiomes in natural farmland soil
Source: Sci Rep. 2024 Jul 2;14:15096. doi: 10.1038/s41598-024-63254-x (PMC11220033; doi:10.1038/s41598-024-63254-x)
Supplement: Supplementary file 9 — Supplementary Table 5. [file 41598_2024_63254_MOESM9_ESM.docx]

Supplementary Table 5. GO term annotation for sample BNFC

| GO:0008150 | biological process | biological_process | 432 |
| --- | --- | --- | --- |
| GO:0071973 | bacterial-type flagellar cell motility | biological_process | 49 |
| GO:0071840 | cellular component organization or biogenesis | biological_process | 524 |
| GO:0071103 | DNA conformation change | biological_process | 308 |
| GO:0045454 | cell redox homeostasis | biological_process | 165 |
| GO:0065003 | macromolecular complex assembly | biological_process | 40 |
| GO:0016226 | iron-sulfur cluster assembly | biological_process | 73 |
| GO:0017004 | cytochrome complex assembly | biological_process | 152 |
| GO:0008152 | metabolic process | biological_process | 8429 |
| GO:0008218 | Bioluminescence | biological_process | 1 |
| **GO:0009058** | **biosynthetic process** | **biological_process** | **5517** |
| GO:0017000 | antibiotic biosynthetic process | biological_process | 95 |
| GO:0009403 | toxin biosynthetic process | biological_process | 16 |
| GO:0006259 | DNA metabolic process | biological_process | 2175 |
| GO:0016070 | RNA metabolic process | biological_process | 1604 |
| GO:0006351 | transcription, DNA-templated | biological_process | 1030 |
| GO:0005975 | carbohydrate metabolic process | biological_process | 2214 |
| GO:0006091 | generation of precursor metabolites and energy | biological_process | 792 |
| GO:0006629 | lipid metabolic process | biological_process | 1023 |
| GO:0015948 | Methanogenesis | biological_process | 52 |
| GO:0006807 | nitrogen compound metabolic process | biological_process | 5234 |
| GO:0016310 | Phosphorylation | biological_process | 1706 |
| GO:0015979 | Photosynthesis | biological_process | 30 |
| GO:0006508 | Proteolysis | biological_process | 2013 |
| GO:0044281 | small molecule metabolic process | biological_process | 5914 |
| GO:0006412 | Translation | biological_process | 2266 |
| GO:0007155 | cell adhesion | biological_process | 43 |
| GO:0006457 | protein folding | biological_process | 208 |
| GO:0007165 | signal transduction | biological_process | 872 |
| GO:0000160 | phosphorelay signal transduction system | biological_process | 1752 |
| GO:0009607 | response to biotic stimulus | biological_process | 6 |
| GO:0009628 | response to abiotic stimulus | biological_process | 3 |
| GO:0042221 | response to chemical | biological_process | 222 |
| GO:0006950 | response to stress | biological_process | 883 |
| GO:0009372 | quorum sensing | biological_process | 0 |
| GO:0006282 | regulation of DNA repair | biological_process | 18 |
| GO:0006808 | regulation of nitrogen utilization | biological_process | 22 |
| GO:0019222 | regulation of metabolic process | biological_process | 2899 |
| GO:0009405 | Pathogenesis | biological_process | 12 |
| GO:0043934 | Sporulation | biological_process | 12 |
| GO:0006810 | Transport | biological_process | 6120 |
| GO:0016032 | viral process | biological_process | 7 |
| GO:0046718 | viral entry into host cell | biological_process | 0 |
| GO:0005575 | cellular component | cellular_component | 126 |
| GO:0005576 | extracellular region | cellular_component | 39 |
| GO:0005618 | cell wall | cellular_component | 8 |
| GO:0009276 | Gram-negative-bacterium-type cell wall | cellular_component | 0 |
| GO:0016020 | Membrane | cellular_component | 5001 |
| GO:0019867 | outer membrane | cellular_component | 301 |
| GO:0042597 | periplasmic space | cellular_component | 137 |
| GO:0031224 | intrinsic to membrane | cellular_component | 3977 |
| GO:0019898 | extrinsic component of membrane | cellular_component | 0 |
| GO:0005886 | plasma membrane | cellular_component | 953 |
| GO:0005622 | Intracellular | cellular_component | 234 |
| GO:0005737 | Cytoplasm | cellular_component | 963 |
| GO:1902494 | catalytic complex | cellular_component | 50 |
| GO:0009317 | acetyl-CoA carboxylase complex | cellular_component | 54 |
| GO:0009349 | riboflavin synthase complex | cellular_component | 16 |
| GO:0009346 | citrate lyase complex | cellular_component | 0 |
| GO:0043190 | ATP-binding cassette (ABC) transporter complex | cellular_component | 227 |
| GO:0000015 | phosphopyruvate hydratase complex | cellular_component | 28 |
| GO:0009341 | beta-galactosidase complex | cellular_component | 21 |
| GO:0019008 | molybdopterin synthase complex | cellular_component | 22 |
| GO:1990204 | oxidoreductase complex | cellular_component | 68 |
| GO:0033202 | DNA helicase complex | cellular_component | 14 |
| GO:0042575 | DNA polymerase complex | cellular_component | 76 |
| GO:0098796 | membrane protein complex | cellular_component | 46 |
| GO:0016469 | proton-transporting two-sector ATPase complex | cellular_component | 161 |
| GO:0005839 | proteasome core complex | cellular_component | 20 |
| GO:0005874 | Microtubule | cellular_component | 1 |
| GO:0048500 | signal recognition particle | cellular_component | 25 |
| GO:0009288 | bacterial type flagellum | cellular_component | 37 |
| GO:0005840 | Ribosome | cellular_component | 1256 |
| GO:0005694 | Chromosome | cellular_component | 103 |
| GO:0005727 | extrachromosomal circular DNA | cellular_component | 0 |
| GO:0005634 | Nucleus | cellular_component | 1 |
| GO:0009579 | Thylakoid | cellular_component | 3 |
| GO:0019012 | Virion | cellular_component | 1 |
| GO:0003674 | molecular function | molecular_function | 1175 |
| GO:0016209 | antioxidant activity | molecular_function | 285 |
| GO:0004601 | peroxidase activity | molecular_function | 72 |
| GO:0003824 | catalytic activity | molecular_function | 7100 |
| GO:0016787 | hydrolase activity | molecular_function | 4669 |
| GO:0008233 | peptidase activity | molecular_function | 2387 |
| GO:0016791 | phosphatase activity | molecular_function | 129 |
| GO:0017111 | nucleoside-triphosphatase activity | molecular_function | 2692 |
| GO:0009055 | electron carrier activity | molecular_function | 1193 |
| GO:0016853 | isomerase activity | molecular_function | 1291 |
| GO:0016874 | ligase activity | molecular_function | 2043 |
| GO:0016829 | lyase activity | molecular_function | 1629 |
| GO:0016491 | oxidoreductase activity | molecular_function | 7781 |
| GO:0000150 | recombinase activity | molecular_function | 12 |
| GO:0004803 | transposase activity | molecular_function | 263 |
| GO:0030170 | pyridoxal phosphate binding | molecular_function | 763 |
| GO:0019842 | vitamin binding | molecular_function | 441 |
| GO:0030246 | carbohydrate binding | molecular_function | 122 |
| GO:0046906 | tetrapyrrole binding | molecular_function | 1101 |
| GO:0043167 | ion binding | molecular_function | 21 |
| GO:0005515 | protein binding | molecular_function | 4288 |
| GO:0016597 | amino acid binding | molecular_function | 87 |
| GO:0046872 | metal ion binding | molecular_function | 2642 |
| GO:0008658 | penicillin binding | molecular_function | 184 |
| GO:0050662 | coenzyme binding | molecular_function | 2090 |
| GO:0051536 | iron-sulfur cluster binding | molecular_function | 973 |
| GO:0003676 | nucleic acid binding | molecular_function | 5756 |
| GO:0000166 | nucleotide binding | molecular_function | 8696 |
| GO:0008134 | transcription factor binding | molecular_function | 254 |
| GO:0003700 | transcription factor activity, sequence-specific DNA binding | molecular_function | 1280 |
| GO:0005215 | transporter activity | molecular_function | 3963 |
| GO:0090484 | drug transporter activity | molecular_function | 0 |
| GO:0016740 | transferase activity | molecular_function | 5650 |
| GO:0016301 | kinase activity | molecular_function | 1820 |
| GO:0016779 | nucleotidyltransferase activity | molecular_function | 905 |
| GO:0004871 | signal transducer activity | molecular_function | 729 |
| GO:0004872 | receptor activity | molecular_function | 670 |
| GO:0003735 | structural constituent of ribosome | molecular_function | 1258 |
