# Supplementary material for: Shotgun metagenomic insights into secondary metabolite biosynthetic gene clusters reveal taxonomic and functional profiles of microbiomes in natural farmland soil
Source: Sci Rep. 2024 Jul 2;14:15096. doi: 10.1038/s41598-024-63254-x (PMC11220033; doi:10.1038/s41598-024-63254-x)
Supplement: Supplementary file 10 — Supplementary Table 6. [file 41598_2024_63254_MOESM10_ESM.docx]

Supplementary Table 6. GO term annotation for sample BNFW

| GO:0008150 | biological process | biological_process | 237 |
| --- | --- | --- | --- |
| GO:0071973 | bacterial-type flagellar cell motility | biological_process | 44 |
| GO:0071840 | cellular component organization or biogenesis | biological_process | 301 |
| GO:0071103 | DNA conformation change | biological_process | 171 |
| GO:0045454 | cell redox homeostasis | biological_process | 106 |
| GO:0065003 | macromolecular complex assembly | biological_process | 23 |
| GO:0016226 | iron-sulfur cluster assembly | biological_process | 42 |
| GO:0017004 | cytochrome complex assembly | biological_process | 77 |
| GO:0008152 | metabolic process | biological_process | 5255 |
| GO:0008218 | Bioluminescence | biological_process | 0 |
| **GO:0009058** | **biosynthetic process** | **biological_process** | **3293** |
| GO:0017000 | antibiotic biosynthetic process | biological_process | 49 |
| GO:0009403 | toxin biosynthetic process | biological_process | 9 |
| GO:0006259 | DNA metabolic process | biological_process | 1402 |
| GO:0016070 | RNA metabolic process | biological_process | 927 |
| GO:0006351 | transcription, DNA-templated | biological_process | 727 |
| GO:0005975 | carbohydrate metabolic process | biological_process | 1214 |
| GO:0006091 | generation of precursor metabolites and energy | biological_process | 480 |
| GO:0006629 | lipid metabolic process | biological_process | 629 |
| GO:0015948 | Methanogenesis | biological_process | 44 |
| GO:0006807 | nitrogen compound metabolic process | biological_process | 3189 |
| GO:0016310 | Phosphorylation | biological_process | 942 |
| GO:0015979 | Photosynthesis | biological_process | 13 |
| GO:0006508 | Proteolysis | biological_process | 1121 |
| GO:0044281 | small molecule metabolic process | biological_process | 3644 |
| GO:0006412 | Translation | biological_process | 1545 |
| GO:0007155 | cell adhesion | biological_process | 33 |
| GO:0006457 | protein folding | biological_process | 111 |
| GO:0007165 | signal transduction | biological_process | 492 |
| GO:0000160 | phosphorelay signal transduction system | biological_process | 929 |
| GO:0009607 | response to biotic stimulus | biological_process | 4 |
| GO:0009628 | response to abiotic stimulus | biological_process | 0 |
| GO:0042221 | response to chemical | biological_process | 120 |
| GO:0006950 | response to stress | biological_process | 484 |
| GO:0009372 | quorum sensing | biological_process | 0 |
| GO:0006282 | regulation of DNA repair | biological_process | 6 |
| GO:0006808 | regulation of nitrogen utilization | biological_process | 20 |
| GO:0019222 | regulation of metabolic process | biological_process | 1853 |
| GO:0009405 | Pathogenesis | biological_process | 0 |
| GO:0043934 | Sporulation | biological_process | 5 |
| GO:0006810 | Transport | biological_process | 3628 |
| GO:0016032 | viral process | biological_process | 2 |
| GO:0046718 | viral entry into host cell | biological_process | 0 |
| GO:0005575 | cellular component | cellular_component | 80 |
| GO:0005576 | extracellular region | cellular_component | 22 |
| GO:0005618 | cell wall | cellular_component | 2 |
| GO:0009276 | Gram-negative-bacterium-type cell wall | cellular_component | 0 |
| GO:0016020 | Membrane | cellular_component | 2975 |
| GO:0019867 | outer membrane | cellular_component | 139 |
| GO:0042597 | periplasmic space | cellular_component | 75 |
| GO:0031224 | intrinsic to membrane | cellular_component | 2255 |
| GO:0019898 | extrinsic component of membrane | cellular_component | 0 |
| GO:0005886 | plasma membrane | cellular_component | 581 |
| GO:0005622 | Intracellular | cellular_component | 145 |
| GO:0005737 | Cytoplasm | cellular_component | 545 |
| GO:1902494 | catalytic complex | cellular_component | 32 |
| GO:0009317 | acetyl-CoA carboxylase complex | cellular_component | 40 |
| GO:0009349 | riboflavin synthase complex | cellular_component | 9 |
| GO:0009346 | citrate lyase complex | cellular_component | 0 |
| GO:0043190 | ATP-binding cassette (ABC) transporter complex | cellular_component | 152 |
| GO:0000015 | phosphopyruvate hydratase complex | cellular_component | 17 |
| GO:0009341 | beta-galactosidase complex | cellular_component | 8 |
| GO:0019008 | molybdopterin synthase complex | cellular_component | 5 |
| GO:1990204 | oxidoreductase complex | cellular_component | 48 |
| GO:0033202 | DNA helicase complex | cellular_component | 4 |
| GO:0042575 | DNA polymerase complex | cellular_component | 43 |
| GO:0098796 | membrane protein complex | cellular_component | 32 |
| GO:0016469 | proton-transporting two-sector ATPase complex | cellular_component | 117 |
| GO:0005839 | proteasome core complex | cellular_component | 9 |
| GO:0005874 | Microtubule | cellular_component | 0 |
| GO:0048500 | signal recognition particle | cellular_component | 18 |
| GO:0009288 | bacterial type flagellum | cellular_component | 31 |
| GO:0005840 | Ribosome | cellular_component | 927 |
| GO:0005694 | Chromosome | cellular_component | 52 |
| GO:0005727 | extrachromosomal circular DNA | cellular_component | 0 |
| GO:0005634 | Nucleus | cellular_component | 0 |
| GO:0009579 | Thylakoid | cellular_component | 4 |
| GO:0019012 | Virion | cellular_component | 0 |
| GO:0003674 | molecular function | molecular_function | 760 |
| GO:0016209 | antioxidant activity | molecular_function | 148 |
| GO:0004601 | peroxidase activity | molecular_function | 36 |
| GO:0003824 | catalytic activity | molecular_function | 4152 |
| GO:0016787 | hydrolase activity | molecular_function | 2592 |
| GO:0008233 | peptidase activity | molecular_function | 1319 |
| GO:0016791 | phosphatase activity | molecular_function | 64 |
| GO:0017111 | nucleoside-triphosphatase activity | molecular_function | 1584 |
| GO:0009055 | electron carrier activity | molecular_function | 703 |
| GO:0016853 | isomerase activity | molecular_function | 673 |
| GO:0016874 | ligase activity | molecular_function | 1164 |
| GO:0016829 | lyase activity | molecular_function | 1012 |
| GO:0016491 | oxidoreductase activity | molecular_function | 4835 |
| GO:0000150 | recombinase activity | molecular_function | 8 |
| GO:0004803 | transposase activity | molecular_function | 270 |
| GO:0030170 | pyridoxal phosphate binding | molecular_function | 490 |
| GO:0019842 | vitamin binding | molecular_function | 294 |
| GO:0030246 | carbohydrate binding | molecular_function | 71 |
| GO:0046906 | tetrapyrrole binding | molecular_function | 682 |
| GO:0043167 | ion binding | molecular_function | 12 |
| GO:0005515 | protein binding | molecular_function | 2309 |
| GO:0016597 | amino acid binding | molecular_function | 50 |
| GO:0046872 | metal ion binding | molecular_function | 1623 |
| GO:0008658 | penicillin binding | molecular_function | 120 |
| GO:0050662 | coenzyme binding | molecular_function | 1344 |
| GO:0051536 | iron-sulfur cluster binding | molecular_function | 582 |
| GO:0003676 | nucleic acid binding | molecular_function | 3769 |
| GO:0000166 | nucleotide binding | molecular_function | 5106 |
| GO:0008134 | transcription factor binding | molecular_function | 107 |
| GO:0003700 | transcription factor activity, sequence-specific DNA binding | molecular_function | 872 |
| GO:0005215 | transporter activity | molecular_function | 2301 |
| GO:0090484 | drug transporter activity | molecular_function | 0 |
| GO:0016740 | transferase activity | molecular_function | 3273 |
| GO:0016301 | kinase activity | molecular_function | 1006 |
| GO:0016779 | nucleotidyltransferase activity | molecular_function | 554 |
| GO:0004871 | signal transducer activity | molecular_function | 402 |
| GO:0004872 | receptor activity | molecular_function | 371 |
| GO:0003735 | structural constituent of ribosome | molecular_function | 928 |
